# C/EBPβ increases tumor aggressiveness by enhancing KIFC1 expression in androgen receptor negative triple negative breast cancer

**DOI:** 10.1186/s12964-025-02243-7

**Published:** 2025-05-30

**Authors:** Shriya Joshi, Chakravarthy Garlapati, Thi Nguyen, Shaligram Sharma, Darshan Shimoga Chandrashekar, Shristi Bhattarai, Sooryanarayana Varambally, Uma Krishnamurti, Xiaoxian Li, Ritu Aneja

**Affiliations:** 1 Department of Biology, Georgia State University, Atlanta, GA 30303 USA; 2 Alkermes Inc, Waltham, MA 02451 USA; 3 Discovery and Development Sciences, Leads Discovery and Optimization, Bristol Myers Squibb, Cambridge, MA 02141 USA; 4 Department of Pathology & Laboratory Medicine, Emory University School of Medicine, Atlanta, GA 30322 USA; 5 Department of Pathology, University of Alabama at Birmingham, Birmingham, AL 35233 USA; 6 Department of Nutrition Sciences, School of Health Professions, University of Alabama at Birmingham, Birmingham, AL 35233 USA; 7 O’Neal Comprehensive Cancer Center, University of Alabama at Birmingham, Birmingham, AL 35233 USA; 8 Department of Pathology, Yale University, New Haven, CT 06510 USA; 9 Chemical Insights Research Institute of UL Research Institutes, Marietta, GA 30067 USA

**Keywords:** AR-TNBC, Proliferation, EMT, AR, C/EBPβ, KIFC1, CW069

## Abstract

Quadruple-negative breast cancers (also known as AR-triple negative (TN) BC) lack the expression of estrogen receptor (ER), progesterone receptor (PR), human epidermal growth factor receptor 2 (HER2), and androgen receptor (AR). AR-TNBC exhibits aggressive characteristics and a poor prognosis. Because of the lack of expression of therapeutic targets, limited therapeutic options exist for patients with AR-TNBC. Hence, new therapeutic targets and risk-predictive biomarkers are required for patients with AR-TNBC. In this study, we investigated the role of kinesin-like protein 1 (KIFC1) in AR-TNBC. We found that C/EBPβ binds to the KIFC1 promoter and induces its expression in the AR-TNBC cells. Notably, AR status was negatively correlated with KIFC1 levels. We also found that AR transcriptionally repressed the transcription factor C/EBPβ, which regulates the expression of KIFC1. The lack of AR expression in AR-TNBC led to C/EBPβ upregulation, thereby enhancing KIFC1 expression. Moreover, upregulation of KIFC1 in AR-TNBC increased cancer cell proliferation and promoted epithelial-mesenchymal transition (EMT), contributing to the aggressive characteristics of AR-TNBC. Inhibiting KIFC1 using the small molecule inhibitor CW069 significantly reduced tumor volume in mice bearing AR-TNBC xenografts, but not in those with triple-negative breast tumors. These data suggest that upregulation of C/EBPβ and KIFC1 contributes to the aggressive characteristics and poor prognosis of AR-TNBC, providing strong evidence that targeting KIFC1 using kinesin inhibitors could be a viable therapeutic approach for patients with AR-TNBC.

**Graphical Abstract:**

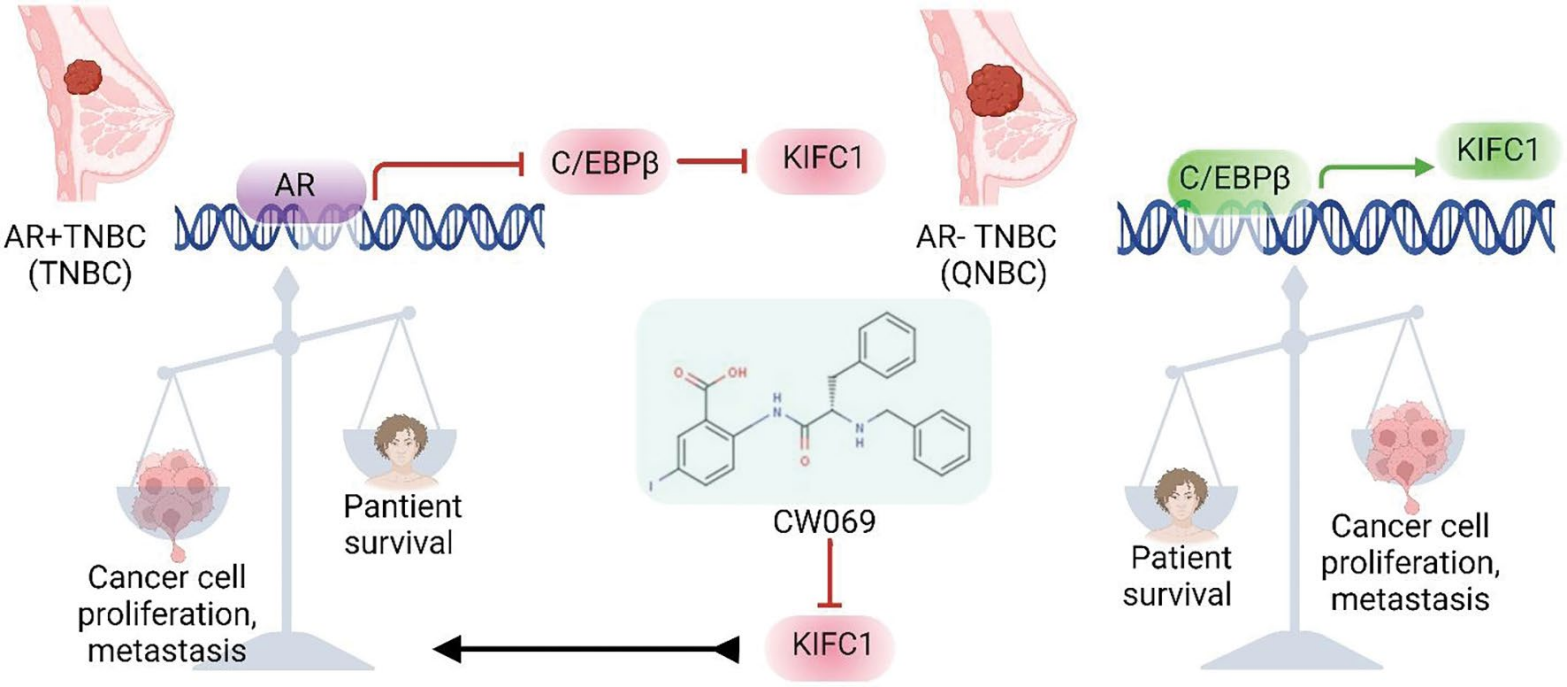

**Supplementary Information:**

The online version contains supplementary material available at 10.1186/s12964-025-02243-7.

## Introduction

Triple-negative breast cancer (TNBC) (hereafter referred to as AR + TNBC) is an aggressive breast cancer subtype that lacks the expression of estrogen receptor (ER), progesterone receptor (PR), and human epidermal growth factor receptor 2 (HER2). Despite the high complexity and heterogeneity of TNBC, it has conventionally been considered a single entity [1]. Nevertheless, recent genome-wide profiling analyses have uncovered various subtypes of AR + TNBC. Notably, androgen receptor (AR), a nuclear steroid hormone receptor, shows variable expression patterns in TNBC and is expressed in 10–43% of patients with TNBC. The remaining 57–90% of TNBCs lack the expression of AR and, thus, can be classified as AR-negative TNBC, also referred to as quadruple-negative breast cancer (AR-TNBC) [2]. AR antagonists such as enzalutamide and bicalutamide have demonstrated favorable efficacy in patients with AR-positive TNBC. However, AR antagonists are unsuitable for patients with AR loss, and patients with AR-TNBC have fewer therapeutic options than those with AR-positive TNBC.

Although the prognostic role of AR in breast cancer is controversial, many studies have shown that the lack of AR expression is associated with an aggressive disease course and poor prognosis [3–7]. AR-negative tumors exhibit a higher Ki67 index, lymph vascular invasion, and metastatic potential than AR-positive TNBCs [8, 9]. Additionally, AR-TNBCs exhibit aggressive basal-like phenotypes and distinct molecular signatures [10, 11]. However, there is a dearth of prognostic biomarkers and therapeutic targets for AR-TNBC. The identification of novel actionable molecular targets and signaling pathways in AR-TNBC is required for the development of targeted therapies to improve disease outcomes in patients with AR loss.

CCAAT/enhancer-binding proteins (C/EBPs) are a family of b-ZIP transcription factors that regulate cell proliferation, differentiation, and survival [12]. Recent findings suggest that high levels of C/EBPβ are associated with aggressive breast cancer subtypes such as ER-negative breast cancer [12, 13]. We and others have previously demonstrated that kinesin-like protein 1 (KIFC1), a centrosome-clustering protein, is associated with AR + TNBC aggressiveness [14, 15, 44]. However, the prognostic value of C/EBPβ and KIFC1 in AR-TNBC remains unclear. In this study, we investigated the role of C/EBPβ and KIFC1 in AR-TNBC.

## Materials and methods

### Patient samples

We procured tissue samples from adult (≥ 18 years old) women with histologically confirmed stage I–III AR + TNBC (*n* = 21) and AR-TNBC (*n* = 58) from Emory hospital tissue bank. Patients were treated with adjuvant chemotherapy at the Emory Hospital or Emory Decatur Hospital. This study was approved by the Institutional Review Board (IRB) of Emory University. All procedures were conducted in accordance with the guidelines of the Emory IRB and Winship Clinical and Translational Review Committee. The patient characteristics are provided in Table 1, and reagents and antibodies that were used are listed in Table 2.

**Table 1:** Patient characteristics

	AR+TNBC (*n*=24)	AR-TNBC (*n*=58)
**AR status**		
Positive	21	0
Negative	0	58
Unknown	2	0
**Age**		
≤60	7	26
>60	16	32
**Race**		
White American	15	9
Black American	4	38
Unknown	5	11
**Chemo type**		
Adjuvant	17	40
No treatment	6	18
**Tubule formation**		
≤2	2	9
>2	21	49
**Nuclear pleomorphism**		
≤2	5	7
>2	18	51
**Mitosis**		
≤5	19	49
>5	4	9
**Nottingham score**		
≤5	4	11
>5	19	47
**Nottingham grade**		
1	4	1
2	3	5
≥3	16	52
**Tumor size (cm)**		
≤3	13	43
>3	10	15
**Vital status**		
Alive	19	21
Dead	4	37
**Recurrence**		
Yes	0	5
No	21	45
Unknown	2	8
**Invasion**		
Yes	0	10
No	21	38
Unknown	2	10

**Table 2:** List of reagents and antibodies

Name of reagent/antibody	Catalog no.	Company
Lipofectamine RNAiMAX	13,778,075	ThermoFisher Scientific
KIFC1 siRNA	L-004958-00-0005	Horizon Discovery
KIFC1 antibody	ab172620	Abcam
RNAeasy mini kit	74,104	Qiagen
iScript cDNA synthesis kit	1,708,891	Bio-Rad
SSO advanced universal SYBR green supermix	1,725,271	Bio-Rad
Protease inhibitor cocktail	P8340	Sigma Aldrich
AR antibody	M356201-2	Dako
C/EBPβ antibody	NBP2-37567	Novus biologicals
Androgen Receptor (AR) (NM_001011645) Human Tagged ORF Clone	RC215709	OriGene
β-actin antibody	SC-47,778	Santa Cruz Biotechnology
Goat anti-mouse HRP antibody	SC-2005	Santa Cruz Biotechnology
Goat anti-rabbit HRP antibody	4050-05	SouthernBiotech
ECL kit	32,106	Thermo Scientific
BrdU cell proliferation kit	2750	EMD Millipore
BrdU antibody	ab152095	Abcam
α tubulin antibody	T9026	Sigma Aldrich
CW069	HY-15,857	MedChem Express
ChIP enzymatic kit	9003	Cell signaling technology
C/EBPB siRNA	L-006423-00-0005	Horizon Discovery
1X phosphate buffer saline	MT21040CV	Corning
AR siRNA	L-003400-00-0005	Horizon Discovery
AR ChIP grade antibody	5153	CST
C/EBPB ChIP grade antibody	PA5-27244	Invitrogen
37% paraformaldehyde	252549-500ML	Millipore Sigma
Crystal violet	C0775-25G	Millipore Sigma
Diva 10X antigen retrieval buffer	DV2004LX	Biocare Medical
Ki-67 antibody	CRM325C	Biocare Medical
pHH3 antibody	3130	Biocare Medical
Rabbit HRP antibody	RHRP520L	Biocare Medical
Mouse HRP antibody	MHRP520L	Biocare Medical
Nude (nu/nu) mice	002019	Jackson laboratories
Maxi prep kit	12,165	Qiagen
Lipofectamine LTX plus	15,338,030	Thermo Fisher Scientific
CellTiter-Glo^®^ 2.0 Cell Viability kit	G9242	Promega
Epithelial-Mesenchymal Transition (EMT) Antibody Sampler Kit	9782T	Cell Signaling Technology
Cell Cycle Regulation Antibody Sampler Kit	#9932	Cell Signaling Technology
MUC1 antibody	ab15481	Abcam
MMP-9 antibody	#3852	Cell Signaling Technology
TWIST1 (E7E2G) antibody	#69,366	Cell Signaling Technology
Cyclin B1 antibody	#4138	Cell Signaling Technology
HMGA1 (D6A4) antibody	#7777	Cell Signaling Technology
Cyclin A2 (BF683)	#4656	Cell Signaling Technology
Cyclin E antibody	#4129	Cell Signaling Technology

### Cell culture

The AR + TNBC cell line HCC70 and AR-TNBC cell lines HCC1806 and BT20 were purchased from ATCC. The AR + TNBC cell line MFM223 was purchased from DSMZ. All cell lines were cultured ineither RPMI (HCC70, HCC1806, BT20), or MEM (MFM223) containing 10% fetal bovine serum and 1% antibiotics and maintained at 37 °C in a 5% CO_2_ atmosphere.

### Chromatin Immunoprecipitation (ChIP)

TFBind and PROMO were used to identify AR- and C/EBPβ-binding sites in *C/EBPβ* (gene encoding C/EBPβ) and *KIFC1*. NCBI Primer Blast and Primer 3 were used to design and validate the ChIP primers compatible with quantitative real-time PCR (qRT-PCR). The primer pairs used for ChIP are listed in Table 3. Chromatin was precipitated and digested using Simple ChIP Enzymatic Chromatin IP Kit (Magnetic Beads). In addition to 10 µg of chromatin, 2% of the input and rabbit IgG was used for immunoprecipitation. DNA was purified and quantified, and qRT-PCR was performed using the SsoAdvanced Universal SYBR Green Supermix. The qRT-PCR results wereanalyzed using QIAxcel Advanced System and QIAxcel ScreenGel software.

**Table 3:** List of qRT-PCR primers

Target gene	Forward primer sequence	Reverse primer sequence
*AR*	GTGCTGGACACGACAACAAC	GATCAGGGGCGAAGTAGAGC
*KIFC1*	CGTGCGAGTTCTCTACCCTG	CAGCCTCCTCTCCTTCTCCT
*CEBPβ*	AAGCACAGCGACGAGTACAA	ACAGCTGCTCCACCTTCTTC
*CCND1*	TACCGTTGACTTCCAGGCAC	GACAGACAAAGCGTCCCTCA
*CCNA2*	ACCCAGGGTTCTCAGAATGG	CTTGGATGCCAGTCTTACTCA
*CDK6*	CCGACTGACACTCGCAGC	GACTTCGGGTGCTCTGTACC
*CDK4*	TGAAATTGGTGTCGGTGCCT	TGGTCGGCTTCAGAGTTTCC
*CDH1*	TGGTACCTGGCAAGATGCAG	GGGGGCTTCATTCACATCCA
*Vim*	GGACCAGCTAACCAACGACA	AAGGTCAAGACGTGCCAGAG
*Snai1*	CGAGTGGTTCTTCTGCGCTA	GGGCTGCTGGAAGGTAAACT
*Slug*	CTCCTCATCTTTGGGGCGAG	TCCTTGAAGCAACCAGGGTC
*Twist*	CGGCCAGGTACATCGACTTC	CAGAGGTGTGAGGATGGTGC
*Zeb1*	GCTGTTTCAAGATGTTTCCTTCCA	TTACACCCAGACTGCGTCAC
*MMP9*	TCTATGGTCCTCGCCCTGAA	TTGTATCCGGCAAACTGGCT
*MUC1*	CCTCACAGTGCTTACAGTTGTT	AGTAGTCGGTGCTGGGATCT
ARE-*C/EBPβ* proximal (for ChIP)	GGCCGCCCTTATAAATAACC	TATTAGTGAGGGGGCTGGTG
ARE*-MYC*-ChIP	GCTCTGGGCACACACATTGG	GGCTCACCCTTGCTGATGCT
CEBPB-RE *KIFC1*		
(-598) ChIP	CCAGCAGTGTGACCTTATTGTG	CGAGCTCGGGGAAGATTTACT

### Plasmid constructs

Customized plasmid constructs for the dual-luciferase reporter assay were purchased from OriGene as bacterial stabs and were used for transfection or co-transfection. The following plasmid constructs were used:

Construct p1 Androgen Receptor (AR) (NM_000044) Human Tagged ORF Clone.

Construct p2 CEBP-beta (C/EBPβ) (NM_005194) Human Tagged ORF Clone.

Construct p3 pRL Renilla Luciferase Control Reporter Vectors.

Construct p4 customized pCMV-Luc Firefly Luciferase mammalian expression reporter vector with C/EBPβ (p4: a) or KIFC1 (p4: b) promoter.

### Dual-luciferase reporter assay

AR + TNBC and AR-TNBC cells were cultured at a density of 2 × 10^4^ cells/well in 96-well plates. Cells were transfected with the constructs p1, p2, AR siRNAs, or C/EBPβ siRNAs on day 1. On day 2, cells were co-transfected with 0.2 µg of the luciferase reporter construct p4 and the internal control vector p3 at a ratio of 10:1 (reporter construct-to-control vector) using Lipofectamine 3000 according to the manufacturer’s protocol. Luciferase activity was measured 48 h post-transfection using the Dual-Luciferase Reporter Assay System (Promega). Renilla luciferase activity was normalized to firefly luciferase activity in cells co-transfected with the reporter construct p4 and the control vector p3.

### Cell transfection

Cells at 70–80% confluency were trypsinized and seeded for transfection with small interfering RNAs (siRNAs) targeting *AR*, *KIFC1*, and *C/EBPβ*; transfection was performed according to the manufacturer’s guidelines. Knockdown (KD) efficiency was assessed 36 h after transfection by immunoblotting. Cells with > 90% KD efficiency were selected for further analysis. Cells were also transfected with AR ORF plasmid DNA (commercially available), according to the manufacturer’s instructions.

### qRT-PCR

mRNA from AR + TNBC and AR-TNBC cells was extracted using the RNeasy kit (QIAGEN) according to the manufacturer’s instructions. iScript cDNA Synthesis Kit was used to generate cDNA, and qRT-PCR was performed using SsoAdvanced Universal SYBR Green Supermix. β-actin was used as a reference gene. The primer pairs used for qRT-PCR are listed in Table 3.

### Immunoblotting

Immunoblotting was performed as previously described [17]. Primary antibodies against KIFC1, cyclin D1, cyclin E, cyclin A, CDK4, CDK6, cyclin B1, SNAI1, MMP9, Muc1, Zeb1 Twist, Slug, and β-actin were used. Goat anti-mouse or goat anti-rabbit IgG horseradish peroxidase (HRP) secondary antibodies were used, and the signal was visualized using an ECL kit. Protein levels were quantified using ImageJ software and normalized to their respective β-actin controls.

### Cell titer Glo (CTG) luminescent cell viability assay

To determine the IC-50 value of the KIFC1 inhibitor CW069, we seeded AR + TNBC and AR-TNBC cells (~ 5 × 10^3^ cells/well in 96-well plates) and treated them with various concentrations of CW069. After incubation, the CTG reagent was added to the cells, and the plates were incubated in a microplate shaker at room temperature for 30 min. Luminescence was recorded using the ID2 SpectraMax, and the data were analyzed using GraphPad Prism 9.0.

### Cell proliferation assay and Immunofluorescence

Cell proliferation was evaluated using the BrdU assay. KIFC1 KD, C/EBPβ KD, CW069-treated, and control cells were used (5 × 10^3^ cells/well in 96-well plates). Cells were incubated with BrdU for 4 h, and BrdU incorporation was measured spectrophotometrically at 450 nm using TMB substrate. For immunofluorescence, cells were incubated with BrdU for 4 h at 37ºC. Cells were fixed in formaldehyde and subjected to acid hydrolysis followed by neutralization with borate buffer. After blocking, cells were incubated with primary antibodies (a cocktail of BrdU and α-tubulin antibody). Cells were incubated with a rabbit and mouse fluorescent secondary antibody cocktail at 37ºC for 40–45 min. Nuclei were counterstained with Hoechst, and coverslips were mounted with ProLong Gold Antifade. Images were acquired using a confocal microscope (LSM 700; ZEISS) and analyzed using ImageJ software.

### Boyden chamber invasion assay

KIFC1 KD, C/EBPβ KD, CW069-treated, and control cells were mixed with serum-free media and seeded on inserts with 8 µM pores in 24-well plates (seeding density 2 × 10^5^ per well). The plates were then incubated for 12–18 h at 37ºC. Boyden chambers were fixed with 3.7% paraformaldehyde and stained with 4% crystal violet. Five different fields were observed for each sample, purple cells were counted independently by two observers, and the mean colony count was determined. Images were acquired using ToupView software.

### Scratch wound migration assay

KIFC1 KD, C/EBPβ KD, CW069-treated, and control cells were seeded in 6-well plates (initial seeding density 2 × 10^6^ cells per well), and a wound was scratched gently. Images were obtained from six different fields using a ZEISS Primovert inverted phase-contrast microscope. Images were acquired at 0 h and 24 h, and wound closure and migration efficiency were analyzed using ImageJ and Adobe Photoshop.

### Immunohistochemistry

Formalin-fixed paraffin-embedded (FFPE) tissue Sect. (5 μm) were deparaffinized and rehydrated in serial ethanol solutions, as previously described [18]. Antigen retrieval was achieved by incubation in Diva 1X buffer (pH 6.0) in a pressure cooker for 10 min at high pressure. Ki-67, pHH3, AR, C/EBPβ, KIFC1, E-cadherin, and vimentin. Mach2 mouse/rabbit HRP antibody was used for the enzymatic detection of the primary antibodies. The biomarkers were reviewed and scored by two independent pathologists. Samples with ≥ 1% AR-positive nuclei were considered AR-positive TNBC. The intensity of staining (none = 0, low = 1, moderate = 2, and high = 3) and percentage of positive cells were determined, and the scores of the two pathologists were averaged. For KIFC1, weighted indices were determined by multiplying the staining intensity score with the percentage of positive cells. For C/EBPβ, H-score was determined.

### Xenograft animal model

Nude female mice were used to establish xenograft animal models. All protocols were in accordance with the guidelines of the Institutional Animal Care and Use Committee. To determine the number of animals required for the study, we performed power analysis using GraphPad Prism 9. MDA-MB-231 (AR + TNBC) and HCC1806 (AR-TNBC) cells were subcutaneously injected into the right flank of mice (4 × 10^6^ cells per flank). When tumors reached 100 mm^3^, mice were divided into vehicle (*n* = 10) and CW069 (*n* = 10) (40 mg/kg) treatment groups. CW069 was administered intraperitoneally twice a week for 28 days. Tumor growth was measured once per week using Vernier calipers, and body weight was recorded for up to 4 weeks. Tumor volume was calculated as length × (width)^2^ ÷ 2. All mice were euthanized at the end of the experiment, and tumors were collected and fixed in 10% formalin. FFPE blocks were prepared, and tissue Sect. (5 μm) were stained with H&E to confirm the tumor area. Six mice per group were monitored for survival for 90 days after xenografting. Fresh-frozen tumor sections from euthanized mice were used for lysate preparation using a BeadBlaster homogenizer.

### Statistical analysis

All experiments were performed in triplicates. Statistical significance was determined using a two-tailed unpaired Student’s *t*-test with Welch’s correction, unpaired nonparametric Mann-Whitney or Kolmogorov-Smirnov test, or one or two-way ANOVA with Tukey’s test for multiple comparisons. Survival data were analyzed using the Mantel-Cox test, and Pearson’s coefficient was used to determine correlations among variables. Data were expressed as mean ± standard error of the mean (SEM) or standard deviation (SD). Statistical analyses were performed using GraphPad Prism version 9.

## Results

### KIFC1 levels are negatively correlated with AR status and are increased in AR-TNBC

**Fig. 1:**
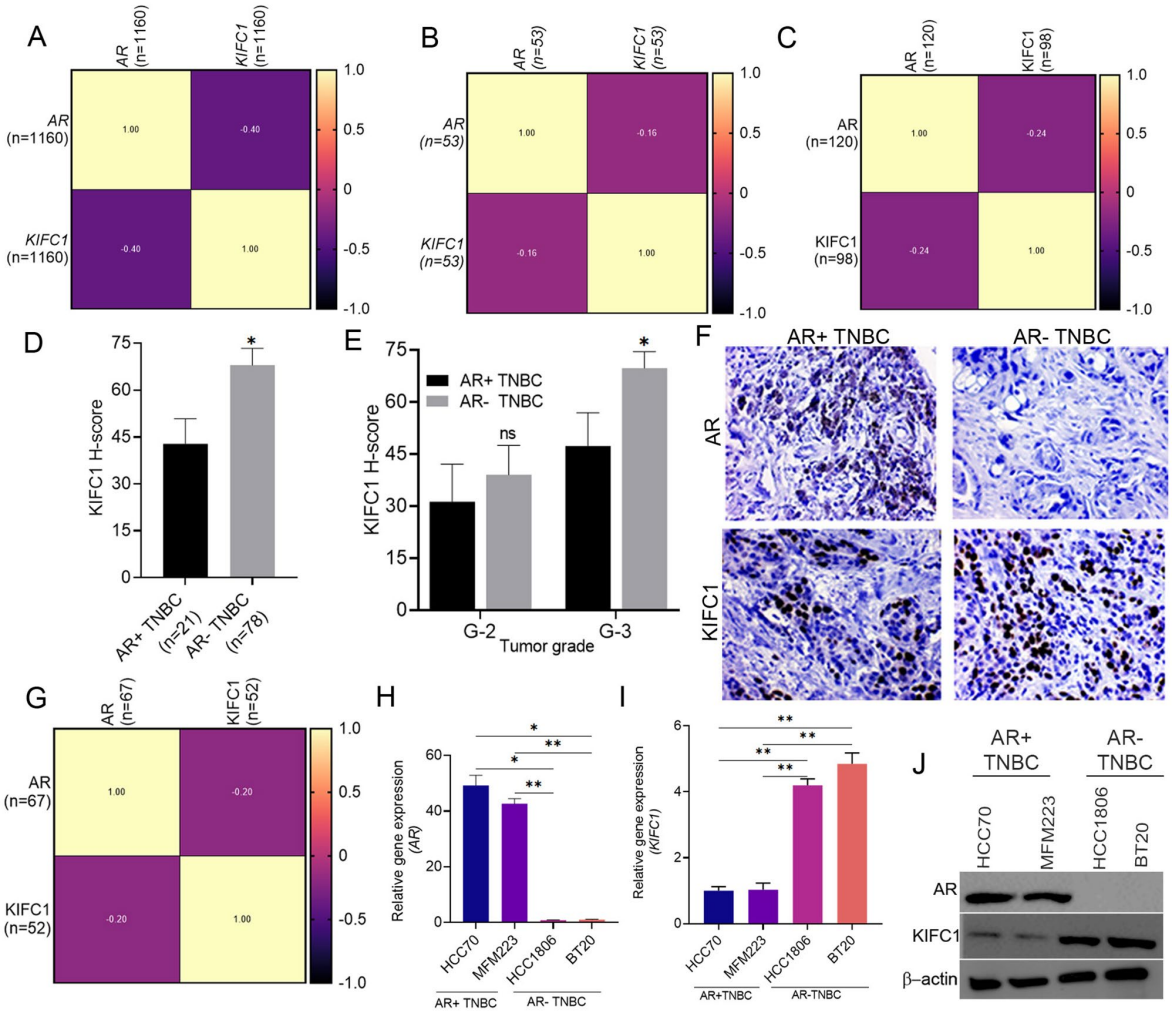
KIFC1 is upregulated in AR-TNBC, and its levels are negatively correlated with AR expression. (**A**–**C**, **G**) Correlation matrix of AR and KIFC1 in cohorts from TCGA (**A**), CCLE (**B**), Emory Hospital (**C**), and Emory Decatur Hospital (**G**). (**D**–**E**) Bar graphs showing KIFC1 protein levels in patients with AR + TNBC and AR-TNBC regardless of grade (**D**) and in patients with grade 2 or grade 3 tumors (**E**) in the Emory dataset. (**F**) Representative IHC images showing expression of AR and KIFC1 in patients with AR + TNBC (*n* = 21) and AR-TNBC (*n* = 58) in the Emory Decatur cohort. (**H**, **I**) Bar graphs showing the mRNA levels of *AR* (H) and *KIFC1* (I) in AR + TNBC and AR-TNBC cell lines. (**J**) Immunoblots showing the levels of AR and KIFC1 in AR + TNBC and AR-TNBC cell lines. HCC70, MFM223 (AR-positive TNBC); 3, 4: HCC1806, BT20 (AR-negative TNBC). Bars indicate mean ± SEM. Unpaired two-tailed Student’s *t*-test with Welch’s correction was used to determine statistical significance. *P < 0.05, **P < 0.005, ns = non-significant

**Fig. 2:**
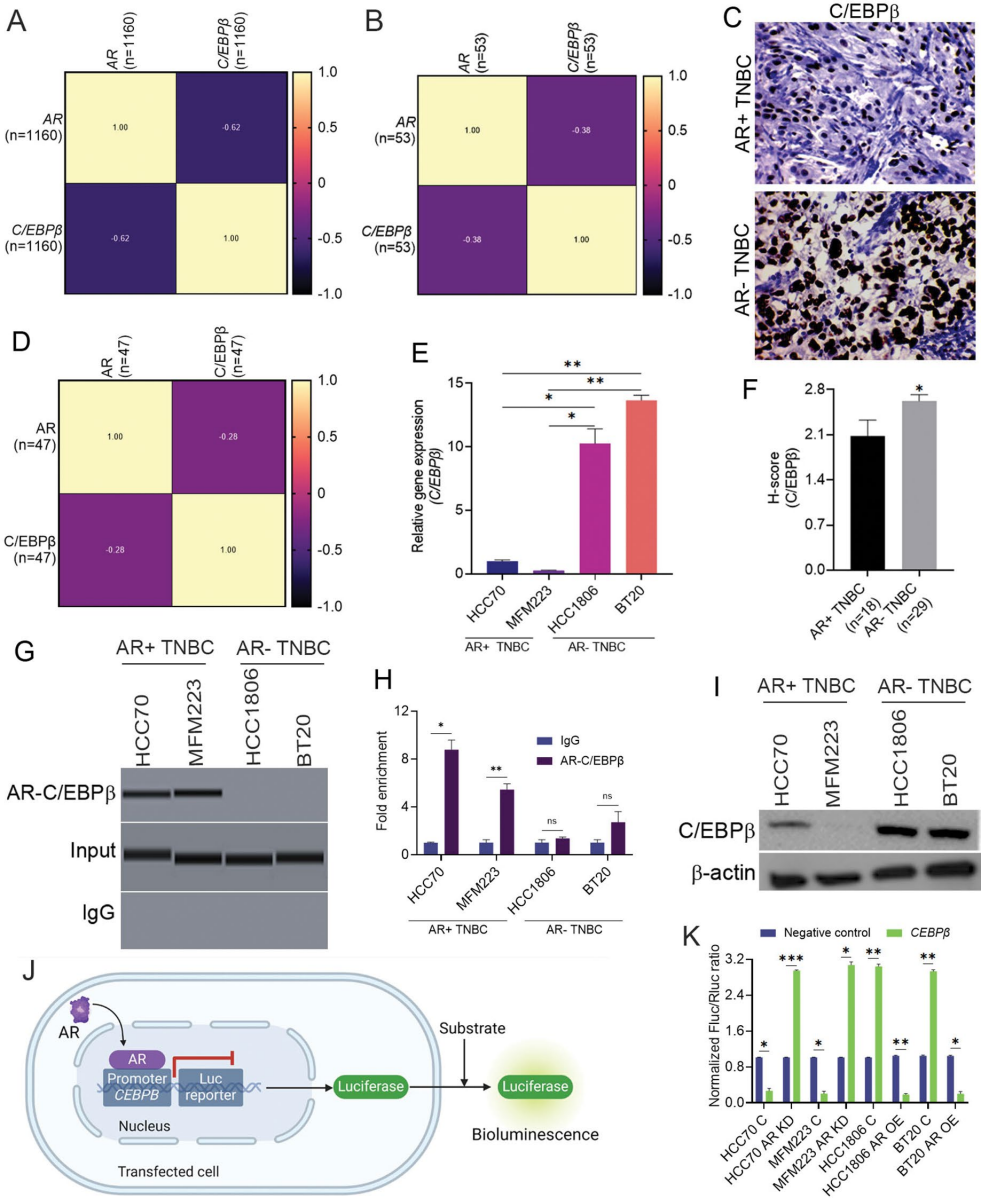
AR may repress *C/EBP*β expression in breast cancer. (**A**–**B**, **D**) Correlation matrix of *AR* and *C/EBPβ* mRNA levels in TCGA (**A**), CCLE (**B**), and Emory Decatur (**D**) datasets. (**C**, **F**) Representative IHC images (**C**) and bar graphs (**F**) showing the expression of C/EBPβ in patients with AR + TNBC (*n* = 21) and AR-TNBC (*n* = 58) in the Emory Decatur cohort. (**G**, **H**) ChIP results showing AR binding to the *C/EBPβ* promoter (**G**). (**E**) Bar graphs showing mRNA levels of *C/EBPβ* in AR + TNBC and AR-TNBC cell lines. (**I**) Immunoblots showing C/EBPβ expression levels in AR + TNBC and AR-TNBC cell lines. (**J**, **K**) Schematic and bar graphs showing luciferase assay in AR + TNBC and AR-TNBC cells. HCC70, MFM223 (AR + TNBC); 3, 4: HCC1806, BT20 (AR-TNBC). Bars indicate mean ± SEM. Unpaired two-tailed Student’s *t*-test with Welch’s correction was used to determine statistical significance. **P < 0.005, ***P < 0.0005, ns = non-significant

We investigated tumor aggressiveness in patients stratified by AR expression and found that AR-TNBC tumors were more aggressive than AR + TNBC tumors (Suppl. Figure 1A–G, Table 1), confirming previous findings [2]. Despite evidence supporting the ability of KIFC1 levels to predict AR + TNBC aggressiveness [14], the role of KIFC1 in AR-TNBC progression remains elusive. We found that AR expression was negatively correlated with KIFC1 levels in various publicly available databases (Fig. 1A, B) and in-house datasets (Fig. 1C, G). We also found that KIFC1 was upregulated in AR-TNBC patients with AR loss compared with those with AR-positive TNBC (Figs. 1D and F and 2F), especially in patients with grade 3 tumors (Fig. 1E). Next, we assessed the expression levels of KIFC1 at the RNA and protein levels in selected AR + TNBC and AR-TNBC cell lines. KIFC1 levels were significantly higher in AR-TNBC than in AR + TNBC cells (Fig. 1H-J). To confirm the relationship between KIFC1 levels and AR status, we silenced AR in AR + TNBC cells and overexpressed (OE) AR in AR-TNBC cells. AR-KD in AR + TNBC cells led to KIFC1 upregulation, whereas AR-OE in AR-TNBC cells resulted in KIFC1 downregulation (Suppl. Figure 2 A, H). In KIFC1 KD cells, AR expression remained unaltered (Suppl. Figure 2G, J). These results suggest that KIFC1 may serve as a novel target in patients with AR-TNBC.

### AR transcriptionally represses C/EBPβ expression

**Fig. 3:**
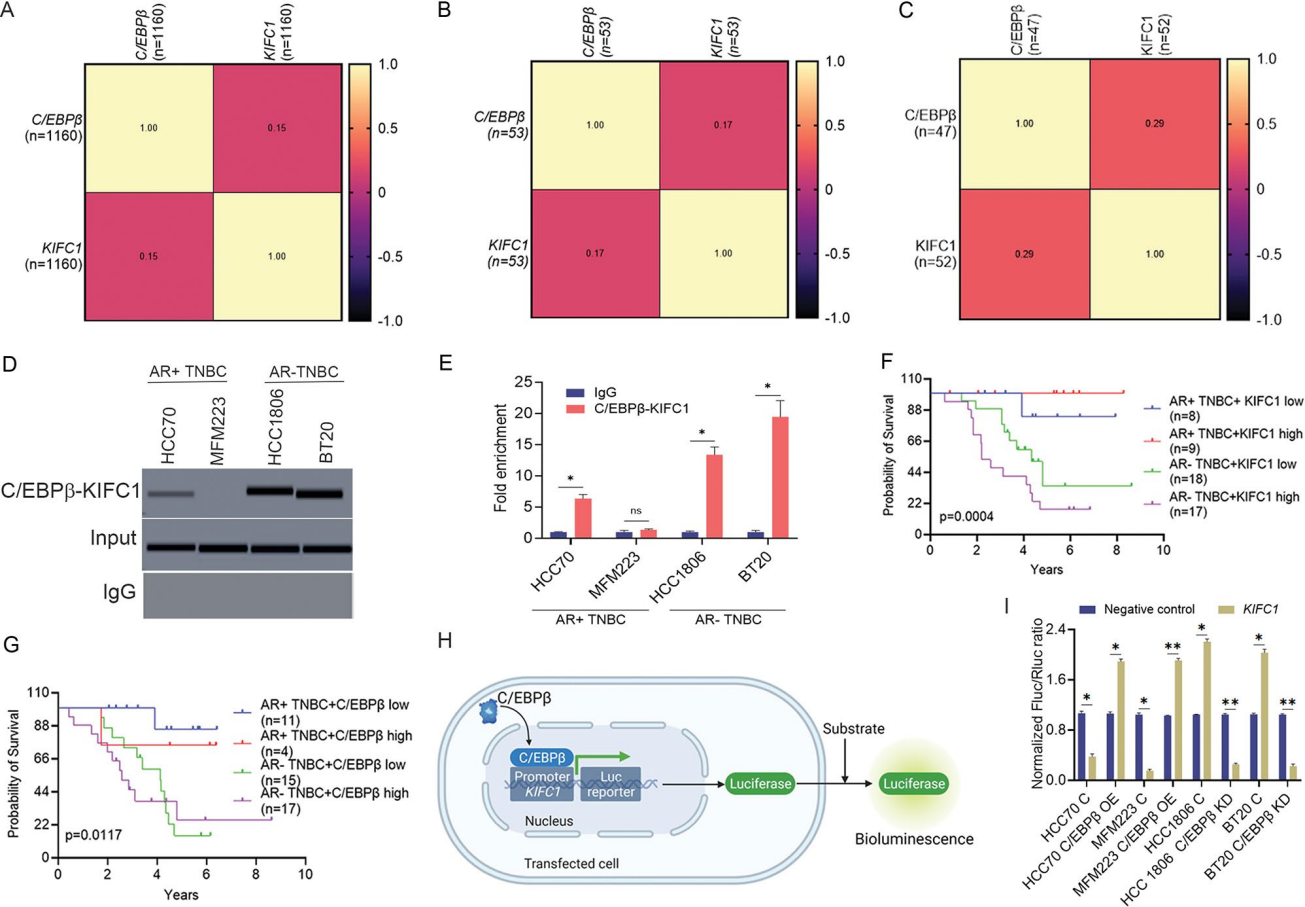
C/EBPβ transcriptionally activates *KIFC1* in breast cancer cells. (**A**–**C**) Correlation matrix of *KIFC1* and *C/EBPβ* mRNA levels in TCGA (**A**), CCLE (**B**), and Emory Decatur (**C**) datasets. (**D**, **E**) ChIP results showing C/EBPβ binding to the *KIFC1* promoter. (**F**, **G**) Kaplan-Meier plots showing the relationship between KIFC1 (**F**) and C/EBPβ (**G**) protein expression levels and survival in patients with AR + TNBC and AR-TNBC in the Emory Decatur cohort. The P-values for survival graphs denote the comparison between high and low protein expression groups (**H**, **I**) Schematic and bar graphs showing luciferase assay in AR + TNBC and AR-TNBC cells. HCC70, MFM223 (AR + TNBC); HCC1806, BT20 (AR-TNBC). Bars indicate mean ± SEM. Unpaired two-tailed Student’s *t*-test with Welch’s correction was to determine statistical significance. *P < 0.05, **P < 0.005

Considering the negative correlation between AR expression and KIFC1 levels, we hypothesized that AR might transcriptionally regulate the expression of *KIFC1*. However, analysis using TFBind failed to identify AR binding sites in the *KIFC1* promoter. Thus, we sought to identify other genes whose expression levels correlated with AR, and found that *C/EBPβ* was one of the most negatively correlated genes (Pearson correlation coefficient − 0.37) in publicly available and in-house datasets (Table 4; Fig. 3A, B, D). Furthermore, C/EBPβ expression was significantly upregulated in AR-TNBC (Fig. 3C, E, F, I). Our ChIP analysis revealed that AR binds to the *C/EBPβ* promoter, regulating its expression (Fig. 3G, H). Similar C/EBPβ upregulation was evident in AR-KD AR + TNBC cells, whereas *C/EBPβ* downregulation was observed in AR-OE AR-TNBC cells (Suppl. Figure 2A, H), suggesting that AR transcriptionally represses *C/EBPβ* expression. AR expression remained unaltered in cells with C/EBPβ KD (Suppl. Figure 2C, J), suggesting that C/EBPβ does not play a role in AR transcriptional regulation. Furthermore, we performed a dual-luciferase assay to validate the ChIP results and to obtain functional insight into the role of AR in *C/EBPβ* expression. Our results revealed a significant decrease in bioluminescence (Fig. 3J, K) when the plasmid containing the AR ORF (p1) was co-transfected with the reporter plasmid containing the promoter sequence of *C/EBPβ* to which AR binds (cloned upstream of the reporter gene; p3 and p4:a). In contrast, cells co-transfected with AR-siRNA and a reporter plasmid containing the *C/EBPβ* promoter sequence exhibited high luminescence (Fig. 3K). These results indicate that AR in AR + TNBC may repress the transcription of *C/EBPβ*. In contrast, AR is not present in AR-TNBC cells, and C/EBPβ is transcriptionally active.

**Table 4:** Genes negatively correlated with *AR.* Source: UALCAN

Gene	Pearson correlation
*SNRPA*	-0.39
*RPP21*	-0.38
*MIIP*	-0.38
*CEBPB*	-0.37
*POLR2F*	-0.37
*COX4I1*	-0.37
*RPS19*	-0.36
*PSMG3*	-0.36
*LSM7*	-0.35
*SHFM1*	-0.35
*C19orf43*	-0.35
*RPS19BP1*	-0.34
*FAM96B*	-0.34
*FAM100B*	-0.34
*NDUFS5*	-0.34
*DGUOK*	-0.34
*UQCRH*	-0.33
*FAM58A*	-0.33
*PFN1*	-0.33
*MRPS24*	-0.33
*MFSD2B*	-0.33
*SH3BGRL3*	-0.33
*CYBA*	-0.33
*TSPO*	-0.33
*PSMG4*	-0.33
*EIF5A*	-0.33
*RPL13*	-0.33
*PFDN2*	-0.33
*ZNHIT1*	-0.33
*RPLP2*	-0.33
*CCDC107*	-0.33
*NUDT1*	-0.32
*C9orf16*	-0.32
*TUBB6*	-0.32
*SURF2*	-0.32
*C6orf108*	-0.32
*BAX*	-0.32
*TMUB1*	-0.32
*BRI3*	-0.32
*GADD45GIP1*	-0.32
*RPL35*	-0.32
*NDUFA11*	-0.32
*C16orf61*	-0.32
*SRM*	-0.31
*CCDC124*	-0.31
*GUK1*	-0.31
*ATP5J2*	-0.31
*ATAD3B*	-0.31
*ZNF593*	-0.31
*C21orf70*	-0.31
*MUTYH*	-0.31
*PUS1*	-0.31
*RRP1*	-0.31
*UBE2S*	-0.3
*TIMM16*	-0.3
*CTU2*	-0.3
*MPST*	-0.3
*ST20*	-0.3
*C7orf55*	-0.3
*RPS7*	-0.3
*CAMTA1*	-0.3
*UQCRHL*	-0.3
*NCRNA00116*	-0.3
*AURKB*	-0.3
*AURKAIP1*	-0.3
*C9orf142*	-0.3
*ATP6V1F*	-0.3
*CCDC102A*	-0.3
*YDJC*	-0.3
*ATAD3A*	-0.3
*BCL7C*	-0.3
*LIN37*	-0.3
*PLEKHO1*	-0.3
*EXOSC4*	-0.3

### C/EBPβ transcriptionally upregulates *KIFC1* in AR-TNBC

As AR expression is negatively associated with KIFC1 and C/EBPβ levels, we evaluated the relationship between C/EBPβ and KIFC1. We found a positive correlation between *C/EBPβ* and KIFC1 levels in TCGA, CCLE, and in-house datasets (Fig. 2A–C). We also found that C/EBPβ upregulated KIFC1 by inducing its transcription (Fig. 2D-E). To validate ChIP results, we performed a dual-luciferase assay. We found significantly higher levels of bioluminescence in cells co-transfected with a plasmid containing C/EBPβ ORF (p2) and the promoter sequence of *KIFC1* where C/EBPβ binds (cloned upstream of reporter gene: p3 and p4:b) than in cells co-transfected with *C/EBPβ*-siRNA and a reporter plasmid containing the *KIFC1* promoter (Fig. 2H, I). Additionally, C/EBPβ silencing resulted in a significant reduction in KIFC1 levels (Suppl. Figure 2D, J). In contrast, C/EBPβ levels remained unaltered in cells with KIFC1 KD (Suppl. Figure 2G, I). We also assessed the prognostic value of KIFC1 and C/EBPβ in AR + TNBC and AR-TNBC. We found that high levels of KIFC1 and C/EBPβ were associated with poor survival, particularly in patients with AR loss (Fig. 2F, G). Survival outcomes within AR-positive TNBC with high or low protein expression of C/EBPβ or KIFC1 were not statistically significant. These results suggest that upregulation of KIFC1-C/EBPβ in AR-TNBC may contribute to the aggressive phenotypes of this breast cancer subtype.

### Silencing C/EBPβ and KIFC1 reduces cell proliferation in AR-TNBC

**Fig. 4:**
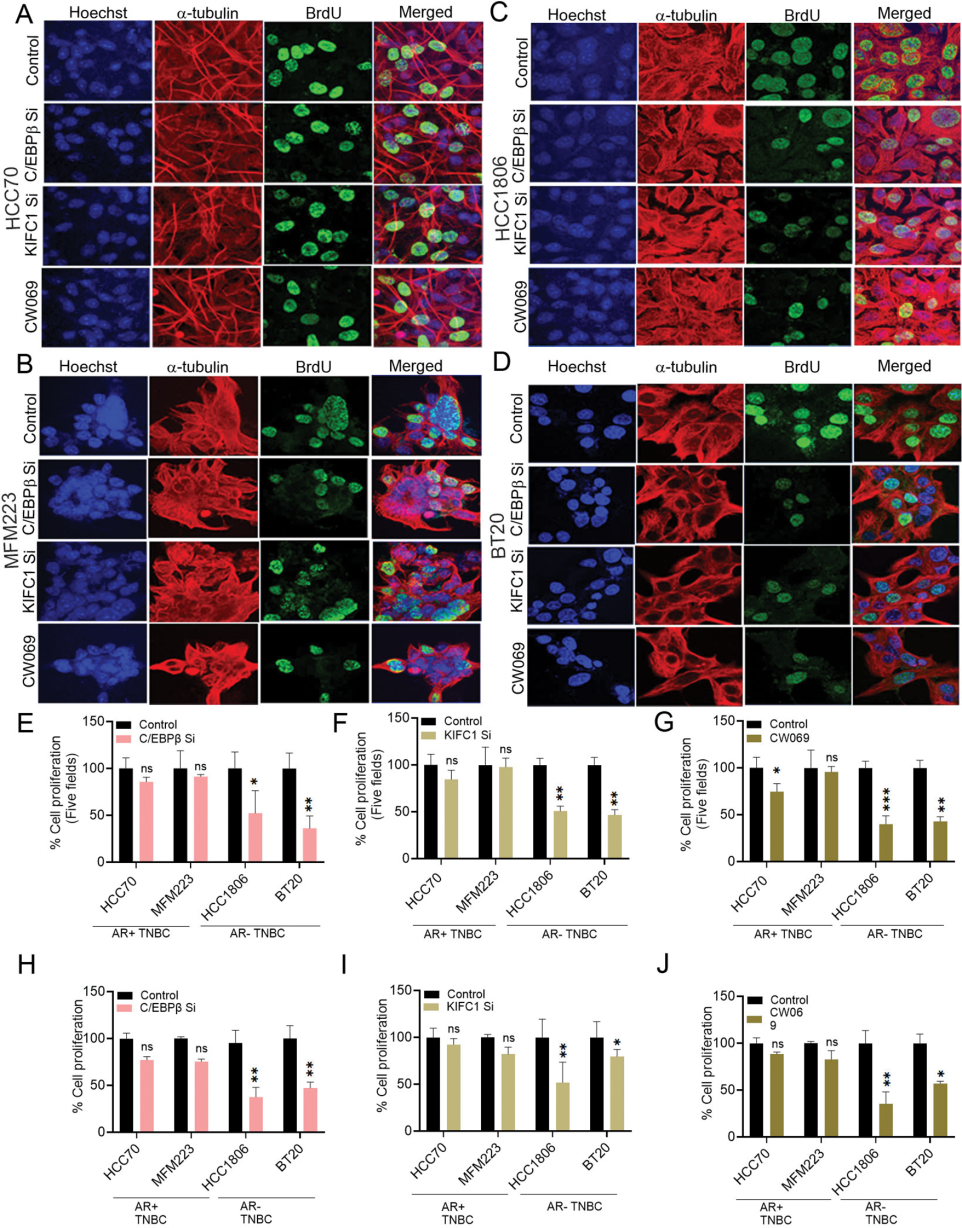
Silencing of C/EBPβ and KIFC1 reduces proliferation in AR-TNBC cells. (**A**–**G**) Representative immunofluorescence images (**A**–**D**) and quantification bar graphs (**E**–**G**) showing BrdU (green) incorporation in AR + TNBC (**A**, **B**) and AR-TNBC (**C**, **D**) cells transfected with scrambled or *C/EBPβ*siRNA (**A**–**D**, **E**) or treated with CW069 (**A**–**D**, **G**). Nuclei were counterstained with Hoechst (blue) and α-tubulin (red). (**H**–**J**) Bar graphs showing BrdU incorporation in cells transfected with scrambled siRNA, *C/EBP* siRNA (**H**), *KIFC1* siRNA (**I**), or treated with CW069 (**J**). Absorbance was measured at 450–540 nm. **A**: HCC70, **B**: MFM223, **C**: HCC1806, **D**: BT20; **A**, **B**-AR-positive TNBC, **C**, D-AR-negative TNBC. Bars indicate mean ± SEM. Unpaired two-tailed Student’s *t*-test with Welch’s correction was used to determine statistical significance. *P < 0.05, **P < 0.005, ns = non-significant

AR-TNBC cells show a higher proliferation rate than AR + TNBC cells [6]. We found that various proliferation-related markers were expressed at higher levels in AR-TNBC than in AR + TNBC cells (Suppl. Figure 3 A-C); hence, we reasoned that upregulation of C/EBPβ and KIFC1 in AR-TNBC may promote cancer cell proliferation. To test this hypothesis, we transfected AR + TNBC and AR-TNBC cells with siRNAs specific for *C/EBPβ* and *KIFC1*, and after confirming the KD efficiency in transfected cells (Suppl. Figure 2B, E, I, J), we performed BrdU cell proliferation assay. AR-TNBC cells were more proliferative at the basal level than AR + TNBC cells. BrdU incorporation was significantly decreased upon silencing of *C/EBPβ* and *KIFC1* in AR-TNBC cells (~ 2 fold); this was less evident in AR + TNBC cells (Fig. 4A–D, E–I). Additionally, treatment of cells with the commercially available KIFC1 inhibitor CW069 (at a concentration less than the IC-50 values; Suppl. Figure 3 J) reduced cell proliferation to a greater extent in AR-TNBC cells (~ 3 fold) than in AR + TNBC cells (~ 1.5 fold) (Fig. 4A–D, G, J). Consistently, *C/EBPβ* and *KIFC1* KD significantly reduced the expression of various proliferative markers in AR-TNBC cells, but not in AR + TNBC cells (Suppl. Figure 3D-I). These results strongly suggest that upregulation of C/EBPβ and KIFC1 contributes to the higher proliferation rate of AR-TNBC cells.

### Silencing C/EBPβ and KIFC1 inhibits cell invasion and migration in AR-negative TNBC

**Fig. 5:**
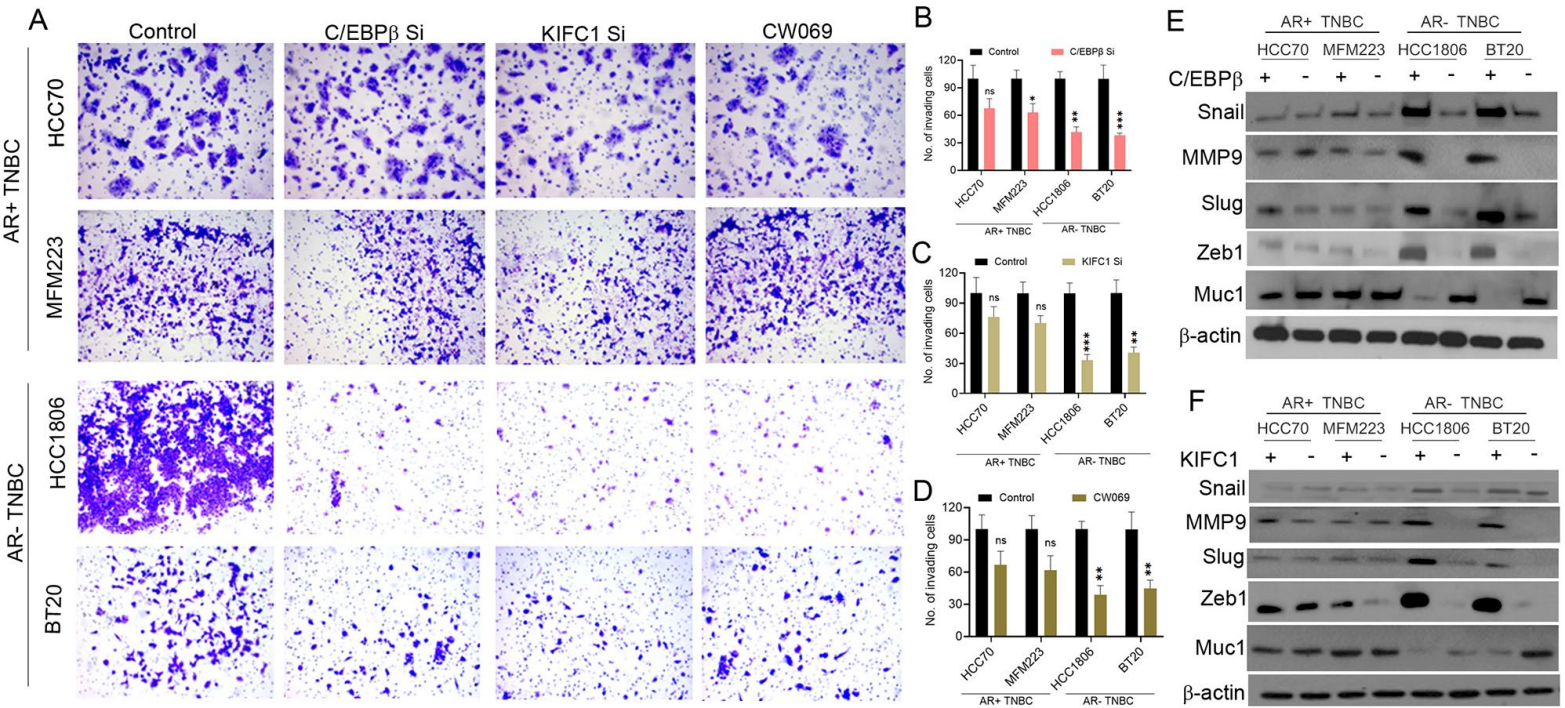
Silencing of KIFC1 and C/EBPβ reduces invasion and EMT in AR-TNBC cells. (**A**–**D**) Representative images (**A**) and bar graphs (**B**-**D**) of control (**A**, **B**–**D**), *C/EBPβ* KD (**A**, **B**), *KIFC1* KD (**A**, **C**), and CW069-treated (**A**, **D**) and AR + TNBC and AR-TNBC cell lines. (**E**, **F**) Immunoblots showing expression levels of various EMT markers in *KIFC1* KD (**E**) and *C/EBPβ* KD in AR + TNBC and AR-TNBC cells (**F**). HCC70, MFM223 (AR + TNBC); HCC1806, BT20 (AR-TNBC). Bars indicate mean ± SEM. Unpaired two-tailed Student’s *t*-test with Welch’s correction was used to determine statistical significance. *P < 0.05, **P < 0.005, ****P < 0.00005, ns = non-significant

**Fig. 6:**
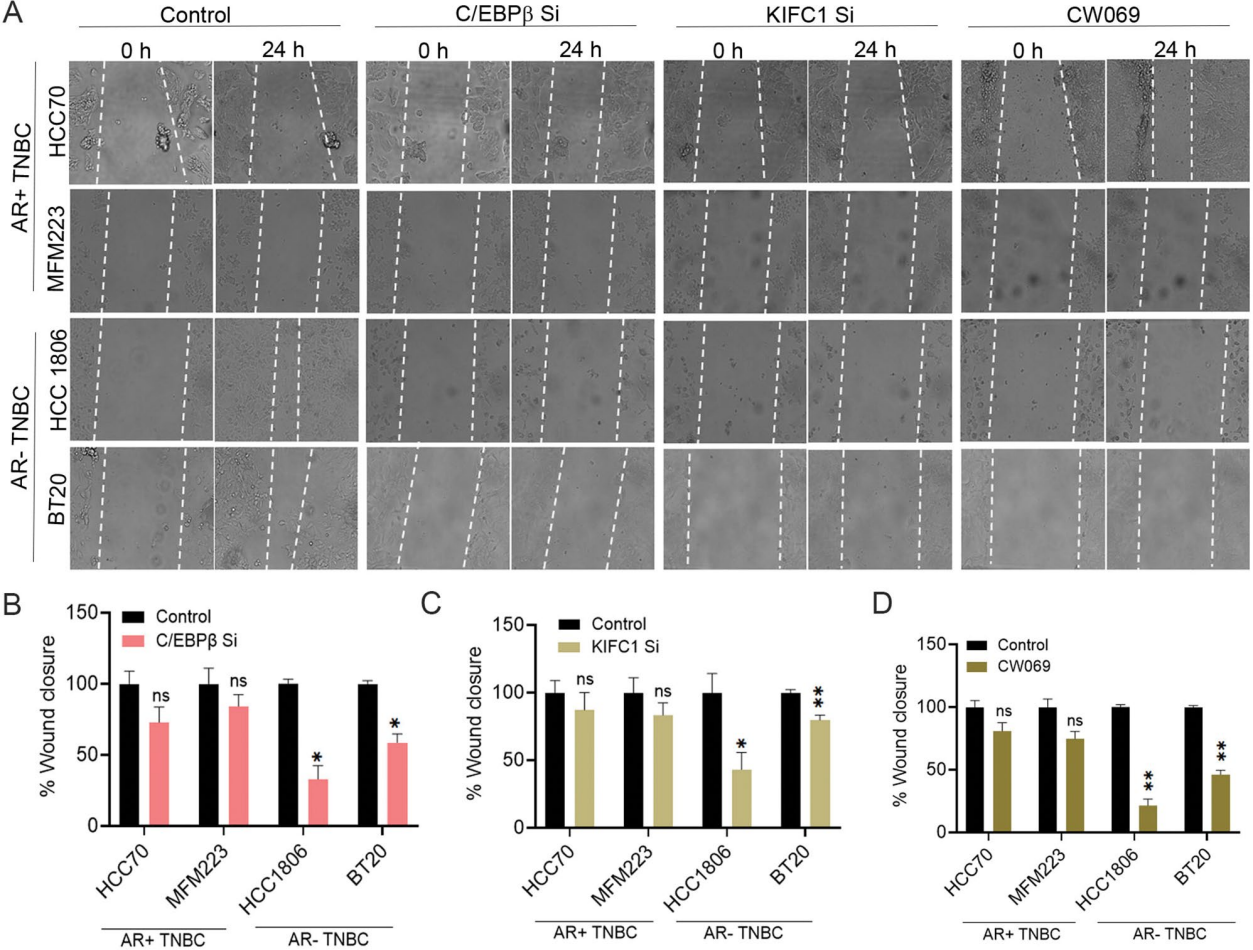
Silencing of KIFC1 and C/EBPβ reduces migration of AR-TNBC cells. (**A**–**D**) Representative images (**A**) and bar graphs (**B**–**D**) of control (**A**, **B**–**D**), C/EBPβ KD (**A**, **B**), KIFC1 LD (**A**, **C**), and CW069-treated (**A**, **D**) AR + TNBC and AR-TNBC cell lines. The dotted lines represent wound closure. Bars indicate mean ± SEM. Unpaired two-tailed Student’s *t*-test with Welch’s correction was used to determine statistical significance. *P < 0.05, **P < 0.005, ns = non-significant

We evaluated the expression of epithelial-mesenchymal transition (EMT) markers in AR + TNBC and AR-TNBC cells and found that various EMT markers were expressed at higher levels in AR-TNBC than in AR + TNBC (Suppl. Figure 4 A–G, J, M). We and others have demonstrated the role of C/EBPβ [20] and KIFC1 [14] in breast cancer cell invasion and migration. To evaluate the role of C/EBPβ and KIFC1 in cell invasion, we silenced their expression and conducted a Boyden chamber invasion assay. AR-TNBC cells were more invasive than AR + TNBC cells at baseline. Silencing C/EBPβ and KIFC1 significantly inhibited cell invasion in AR-TNBC cells, but not in AR + TNBC cells (Fig. 5A–C). Furthermore, inhibition of cell invasion upon CW069 treatment was more prominent in AR-TNBC cells than in AR + TNBC cells (Fig. 5A, D). Consistently, silencing C/EBPβ and KIFC1 reduced the expression levels of various EMT markers in AR-TNBC cells but not in AR + TNBC cells (Fig. 5E, F; Suppl. Figure 4 H–L). Scratch wound migration assays showed that cell migration upon C/EBPβ KD, KIFC1 KD, and CW069 treatment was significantly reduced in AR-TNBC cells but not in AR + TNBC cells (Fig. 6A–D). Collectively, these results suggest that upregulation of C/EBPβ and KIFC1 contributes to the high invasion and migration potential of AR-TNBC cells.

### KIFC1 Inhibition reduces tumor growth in mice bearing AR-TNBC tumors

**Fig. 7:**
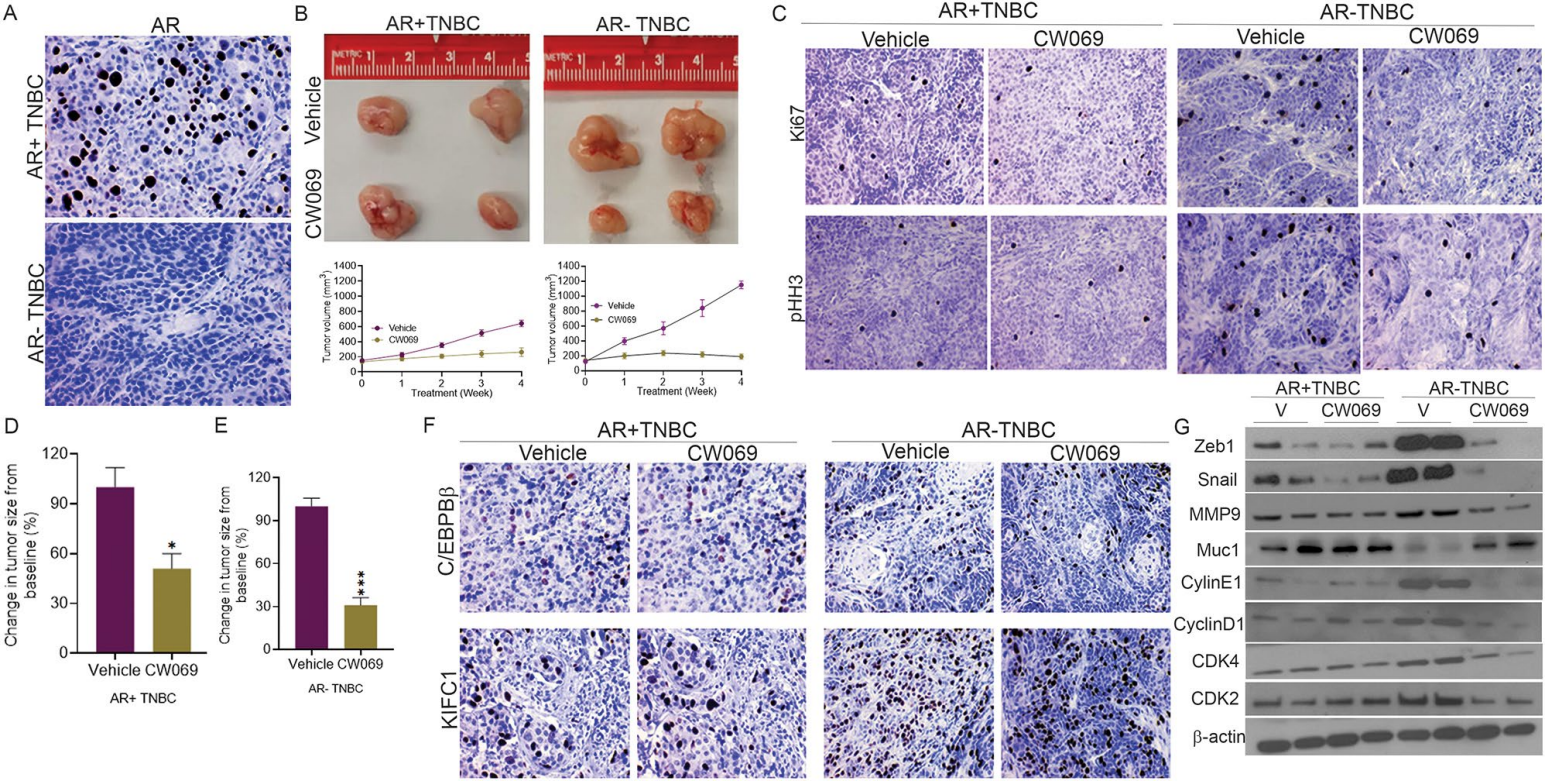
CW069 treatment reduces tumor growth in mice bearing AR-TNBC tumors. (**A**, **C**, **F**) Representative IHC images showing AR (**A**), Ki67 (**C**), pHH3 (**C**), C/EBPβ (**F**), and KIFC1 (**F**) expression levels in mice with AR + TNBC and AR-TNBC tumors. (**B**, **D**, **E**) Representative photographs (**B**) and bar graphs (**B**, **D**, **E**) showing tumor volume in mice bearing AR + TNBC (**B**, **D**) and AR-TNBC (**B**, **E**) tumors after CW069 treatment. (**G**) Immunoblots showing the expression levels of various EMT- and proliferation-related markers in vehicle- and CW069-treated mice bearing AR + TNBC and AR-TNBC xenografts. Vehicle *n* = 10, CW069 *n* = 10 for both AR + TNBC and AR-TNBC xenograft mice. Bars indicate mean ± SEM. Unpaired two-tailed Student’s *t*-test with Welch’s correction was used to determine statistical significance. *P < 0.05, ***P < 0.0005

Our in vitro data suggest that upregulation of C/EBPβ and KIFC1 contributes to the high aggressiveness of AR-TNBC, and that inhibiting KIFC1 could serve as a viable option for patients with AR loss. To validate our hypothesis, we established xenograft mouse models bearing AR + TNBC (MDA-MB-231) and AR-TNBC (HCC1806) tumors (Fig. 7A). Mice were treated with CW069 (*n* = 10) or vehicle (*n* = 10), and tumor growth was assessed for 4 weeks after treatment initiation. We did not observe any differences in the body weight of mice in any of the treatment groups (Suppl. Figure 5 C-D), suggesting an excellent safety profile for CW069. As expected, there was no change in the levels of C/EBPβ, KIFC1, or AR after CW069 treatment in mice bearing AR + TNBC or AR-TNBC xenografts (Fig. 7F; Suppl. Figure 5 A). We also found that CW069 treatment resulted in a profound decrease in the levels of the proliferative markers Ki67 and pHH3 in AR-TNBC but not in AR + TNBC xenografts (Fig. 7C; Suppl. Figure 5B, E). Notably, CW069 treatment resulted in a greater decrease (~ 4 fold) in tumor volume in mice bearing AR-TNBC tumors than in those with AR + TNBC (~ 1.5 fold; (Fig. 7B, D–E). Moreover, the expression levels of various EMT- and proliferation-related markers were significantly reduced in CW069-treated mice bearing AR-TNBC tumors (Fig. 7G). These results provide compelling evidence that KIFC1 inhibition using CW069 could serve as a promising therapeutic approach for the treatment of AR-TNBC.

## Discussion

In contrast to other breast cancer subtypes, AR-TNBC remains underexplored. Although AR-TNBC is often not considered a separate entity, mounting evidence has shown that AR-negative tumors exhibit unique pathobiology, molecular networks, and therapeutic targets [2]. AR-TNBC tumors are characterized by an aggressive disease course [2], a high proliferative index [7], and poor prognosis [21]. Treatment options for patients with AR-TNBC are limited because of the lack of therapeutic targets [22]. Although patients with TNBC benefit from AR antagonists, those with AR-TNBC are not eligible for AR antagonists because their tumors do not express AR [2, 8]. Studies comparing the expression of biomarkers between AR-negative and AR-positive TNBCs are required to identify novel actionable targets unique to AR-TNBC.

Evidence indicates that AR-TNBC tumors are enriched in basal-like and immune subtype-related genes [23], including *C/EBPβ* [24]. C/EBP family members regulate cell proliferation, metabolism, cell differentiation, cell survival, inflammation, oncogene-induced senescence, and tumorigenesis [25–30]. C/EBP proteins, including C/EBPβ, are upregulated in aggressive cancers [13, 19]. Barkat et al. [19] provided compelling evidence that C/EBPβ expression is elevated in castration-resistant prostate cancer compared to benign prostate tumors and localized prostate cancer. They also showed that C/EBPβ was upregulated upon androgen deprivation therapy, and that AR binds to the *C/EBPβ* promoter, inhibiting its transcription. However, the ability of AR to regulate C/EBPβ expression in breast cancer has not been studied. In this study, we found that owing to the lack of AR expression in AR-TNBC, C/EBPβ was upregulated and enhanced the aggressive characteristics of breast cancer cells. In a recent study, Sterken et al. [20] showed that higher C/EBPβ expression was associated with TNBC cell invasion and migration. In their study, they used BT20 cells, which lack AR expression. However, the mechanisms linking AR, C/EBPβ, and cell migration were not investigated. Despite evidence supporting the therapeutic value of C/EBPβ inhibition, C/EBPβ also regulates various non-tumorigenic pathways, and C/EBP proteins often have redundant functions and overlapping binding partners [12]. Thus, target specificity is a major obstacle to the development of C/EBPβ-targeted therapies for AR-TNBC. Furthermore, there is a lack of specificity in the interaction between C/EBPβ and DNA because the CCAAT box motif is universal to all C/EBP family members [12, 31]. This lack of specificity adds a layer of complexity in targeting C/EBPβ and raises the need to identify downstream actionable targets in the AR-C/EBPβ axis.

In this study, we identified KIFC1 as a target downstream of C/EBPβ and found that C/EBPβ regulates KIFC1 and activates its transcription. We previously [14] reported the crucial role of KIFC1 in TNBC aggressiveness. However, the role of KIFC1 in AR-TNBC has remained unclear. To our knowledge, this is the first study to demonstrate a negative correlation between KIFC1 levels and AR expression in breast cancer. Our data suggest that the absence or loss of AR leads to increased C/EBPβ expression in AR-TNBC, resulting in higher expression levels of KIFC1.

KIFC1 is a non-essential gene for normal breast cells; therefore, KIFC1 could serve as a cancer-selective therapeutic target [32–34]. Multiple KIFC1 inhibitors have been developed [35]. The KIFC1 inhibitor CW069 is a small molecule inhibitor with high affinity for the motor domain of KIFC1. By inhibiting the motor domain of KIFC1, CW069 interferes with the ability of KIFC1 to drive the motility of microtubules [22]. CW069 treatment leads to multipolar mitoses in cells with supernumerary centrosomes, causing cancer cell death. In contrast, CW069 treatment does not significantly alter the spindle shape of normal dermal fibroblasts [22]. Despite the high sequence similarity between the motor domains of KIFC1 and kinesin spindle protein (KSP), CW069 selectively inhibits KIFC1 [22], suggesting that CW069 is an attractive drug candidate for the selective inhibition of KIFC1.

The in vitro and in vivo data presented here strongly suggest that inhibition of KIFC1 using CW069 may inhibit cell proliferation and migration to a greater extent in AR-TNBC cells than in AR + TNBC cells. KIFC1-overexpressing cells are resistant to tubulin-targeted drugs, including docetaxel [36]. Thus, targeting the C/EBPβ-KIFC1 axis using CW069 in combination with docetaxel could be a viable approach for improving outcomes in patients with AR-TNBC. Evaluation of the efficacy of this combination for AR-TNBC is currently underway in our laboratory.

This study has certain limitations. Cells express various isoforms of C/EBPβ, including LAP1, LAP2, and LIP. The LIP-to-LAP ratio is a critical determinant of cancer aggressiveness [12, 20]. Although our findings support the crucial role of C/EBPβ in tumor aggressiveness in AR-TNBC, we did not evaluate the role of different C/EBPβ isoforms or the LIP-to-LAP ratio in tumor characteristics. Furthermore, the ability of C/EBPβ to activate transcription is regulated through post-translational modifications. Under physiological conditions, C/EBPβ is maintained in a repressed state by negative regulatory domains, which sterically hinder its transactivation domains [37, 38]. Phosphorylation within the inhibitory domains could revert their repressive effect, leading to the increased transcriptional activity of C/EBPβ [39–43]. Further studies are warranted to assess the effects of various phosphorylation events on the ability of C/EBPβ to activate the transcription of KIFC1 in AR + TNBC and AR-TNBC cells. These studies could provide further insight into differences in the biology of AR + TNBC and AR-TNBC and help develop small molecule inhibitors of kinases for the treatment of patients with AR-TNBC.

In conclusion, our findings highlight for the first time the role of the AR-C/EBPβ-KIFC1 axis in the aggressive features of AR-TNBC. These findings also provide strong evidence that inhibition of KIFC1 using CW069 could serve as a promising therapeutic approach for patients with AR-TNBC. Further clinical validation of the efficacy of CW069 in this patient population is warranted.

## Electronic supplementary material

Below is the link to the electronic supplementary material.

Supplementary Material 1

## Data Availability

No datasets were generated or analysed during the current study.
