# Supplementary material for: C/EBPβ increases tumor aggressiveness by enhancing KIFC1 expression in androgen receptor negative triple negative breast cancer
Source: Cell Commun Signal. 2025 May 30;23:255. doi: 10.1186/s12964-025-02243-7 (PMC12125945; doi:10.1186/s12964-025-02243-7)
Supplement: Supplementary file 1 — Supplementary Material 1 [file 12964_2025_2243_MOESM1_ESM.docx]

**Supplementary figures and legends**


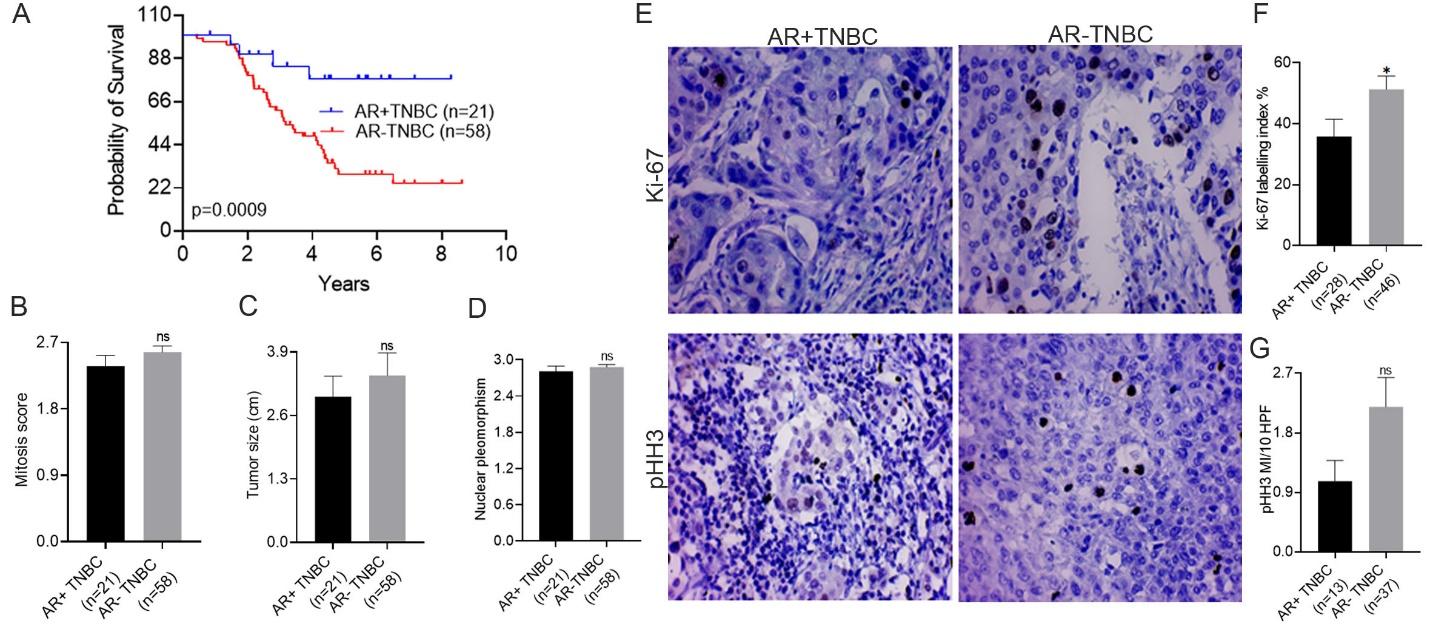


**Suppl. Fig. 1: AR-TNBC is more aggressive than AR+TNBC**. **(A)** Kaplan–Meier curves showing the survival of patients with AR+TNBC and AR-TNBC in the Emory-Decatur dataset. **(B–D)** Bar graphs showing mitosis scores (**B**), tumor size (**C**), and nuclear pleomorphism (**D**) in patients with AR+TNBC and AR-TNBC in the Emory-Decatur dataset. **(E–G)** Representative IHC images (**E**) and their quantitative bar graphs (**F-G**) show the expression of Ki67 (**E, F**) and pHH3 (**E, G**) in patients with AR+TNBC and AR-TNBC. Bars indicate mean ± SEM. Unpaired two-tailed Student’s *t*-test with Welch’s correction was used to determine statistical significance. **P*< 0.05, ns = non-significant.

**
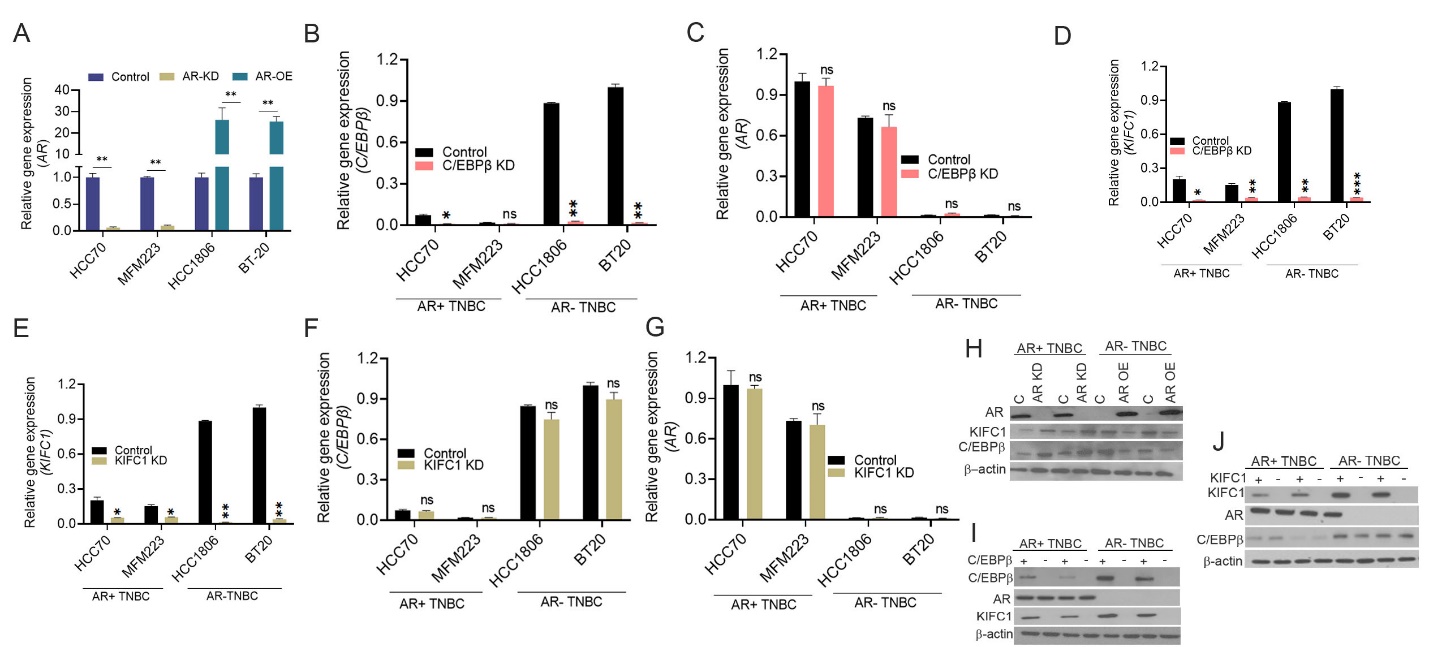
**

**Suppl. Fig. 2: AR transcriptionally downregulates C/EBPβ, and C/EBPβ upregulates KIFC1.** **(A–G)** Bar graphs showing the mRNA levels of *AR* (**A, C, G**), *KIFC1* (**A, D, E**), and *CEBPB* (**A, B, F**) in AR-OE and AR-KD (**A**), *CEBPB*-KD (**B–D**) and *KIFC1*-KD (**E-G**) AR+TNBC and AR-TNBC cells. **(H–J)** Immunoblots showing the levels of AR (**H–J**), KIFC1(**H–J**), and C/EBPB (**H–J**) in AR-KD and AR-OE (**H**), *CEBPB*-KD (**I**), and *KIFC1*-KD (**J**) AR+TNBC and AR-TNBC cells. HCC70, MFM223 (AR+TNBC); HCC1806, BT20 (AR-TNBC). Bars indicate mean ± SEM. Unpaired two-tailed Student’s *t*-test with Welch’s correction was used to determine statistical significance. **P*< 0.05, ***P*< 0.005, ****P*< 0.0005, ns = non-significant.


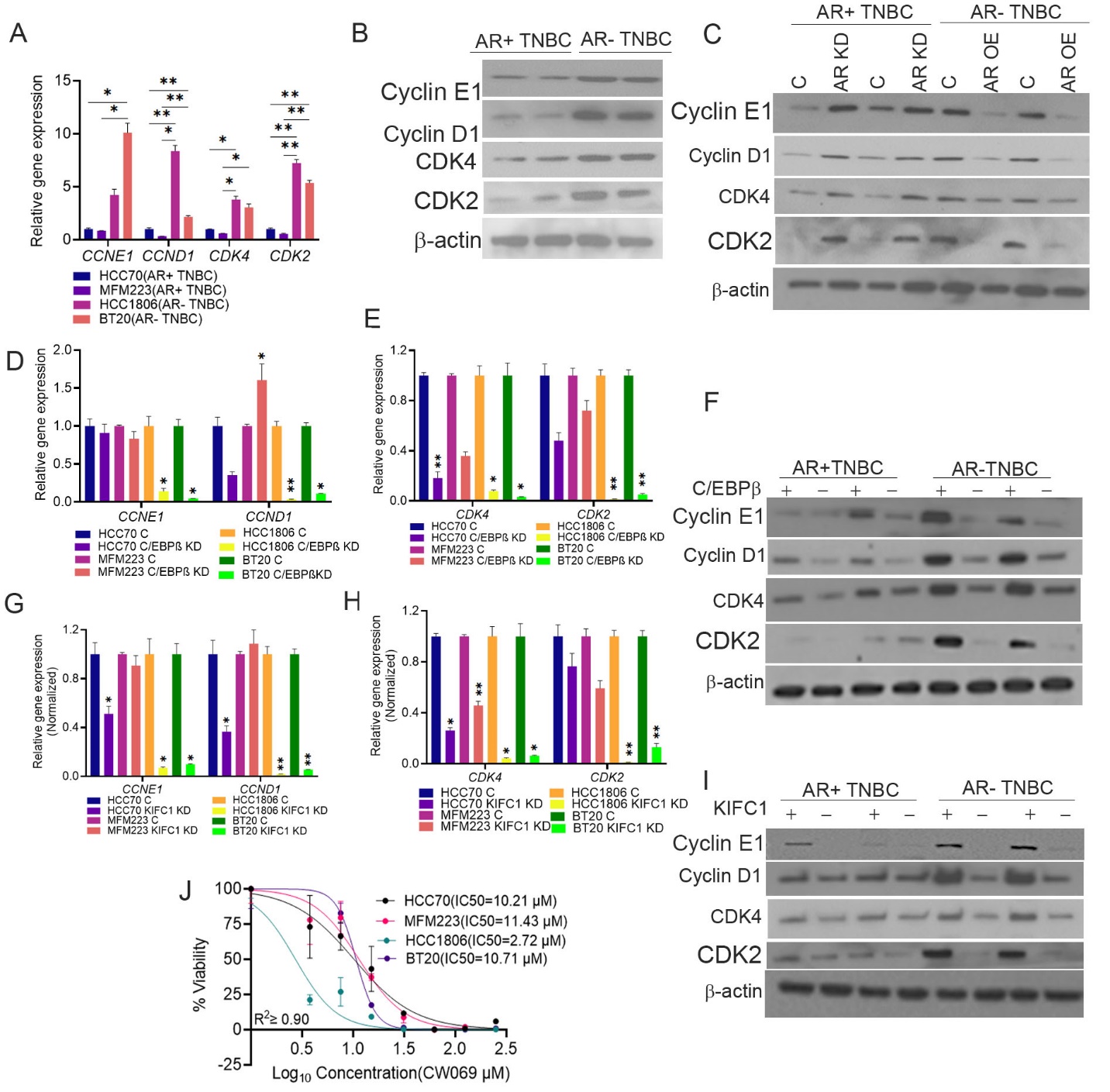


**Suppl. Fig. 3: AR-TNBC cells are more proliferative than AR+TNBC cells.** Bar graphs showing relative gene expression of various proliferation markers in control (**A**), *C/EBPβ*-KD (**D, E**), and *KIFC1*-KD (**G, H**) AR+TNBC and AR-TNBC cells. Immunoblots showing expression of various proliferation markers in control (**B**), *AR*-KD and *AR*-OE (**C**), *C/EBPβ*-KD (**F**), and *KIFC1*-KD (**I**) AR+TNBC and AR-TNBC cells. **(J)** Graph showing IC-50 values (in µM) of CW069 in AR+TNBC and AR-TNBC cells. HCC70, MFM223 (AR+TNBC); HCC1806, BT20 (AR-TNBC). Bars indicate mean ± SEM. Unpaired two-tailed Student’s *t*-test with Welch’s correction was used to determine statistical significance. **P*< 0.05, ***P*< 0.005, ns = non-significant.


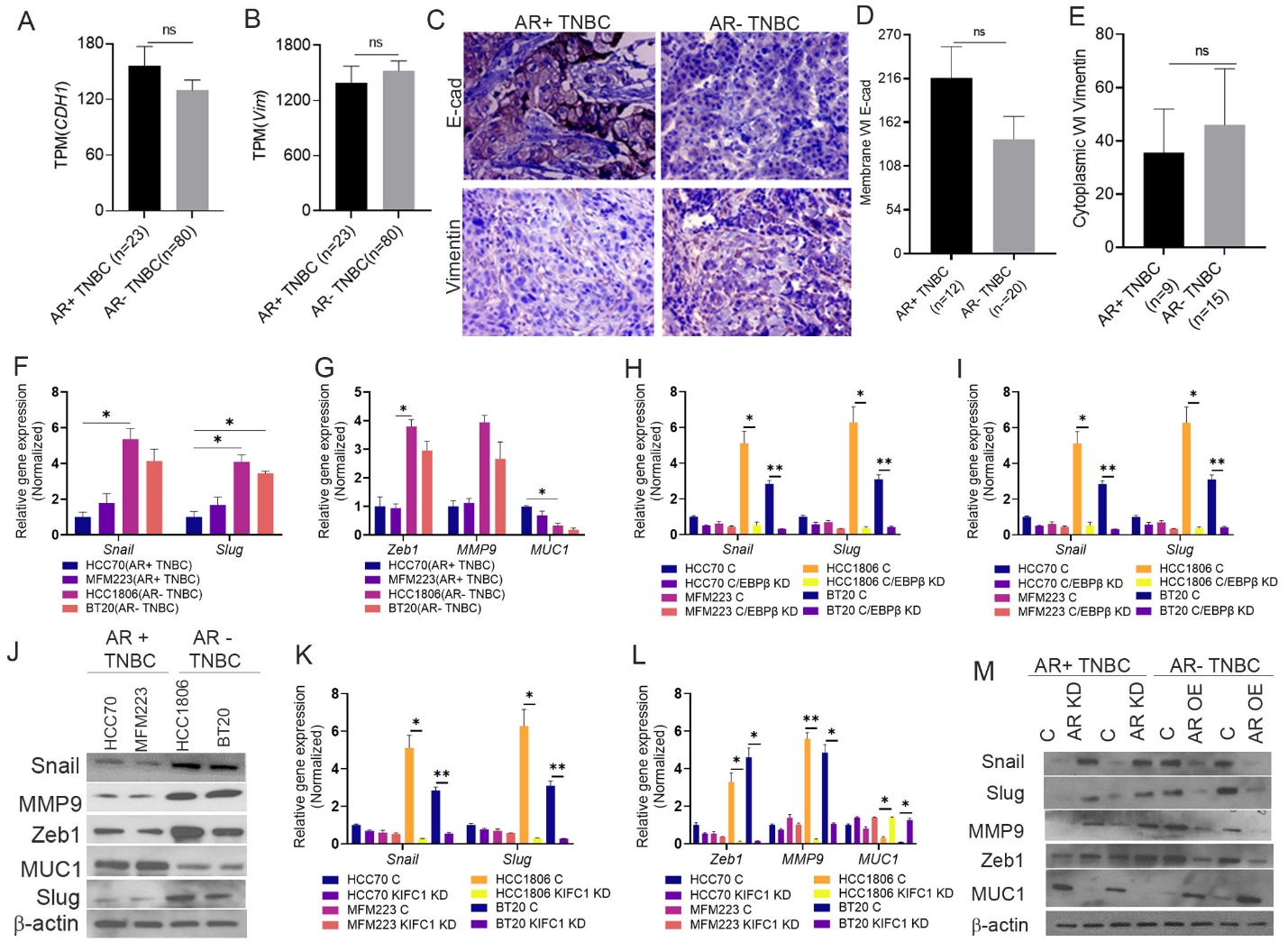


**Suppl. Fig. 4: AR-TNBC cells are more metastatic than AR+TNBC cells**. Bar graphs showing mRNA (**A-B**) and protein (**D-E**) levels of E-cadherin (**A, D**) and vimentin (**B, E**) in TCGA (**A-B**) and Dekalb (**D-E**) datasets. **(C)** Representative IHC images showing expression of E-cadherin and vimentin in patients with AR+TNBC and AR-TNBC. **(F–L)** Bar graphs showing the mRNA levels of various EMT markers in control (**F, G**), *KIFC1*-KD (**H-I**), and *C/EBPβ*-KD (**K-L**) AR+TNBC and AR-TNBC cells. (**J–M**) Immunoblots showing the protein levels of various EMT markers in control, *AR*-KD, and *AR*-OE AR+TNBC and AR-TNBC cells. HCC70, MFM223 (AR+TNBC); HCC1806, BT20 (AR-TNBC). Bars indicate mean ± SEM. Unpaired two-tailed Student’s *t*-test with Welch’s correction was used to determine statistical significance. **P*< 0.05, ***P*< 0.005, ns = non-significant.


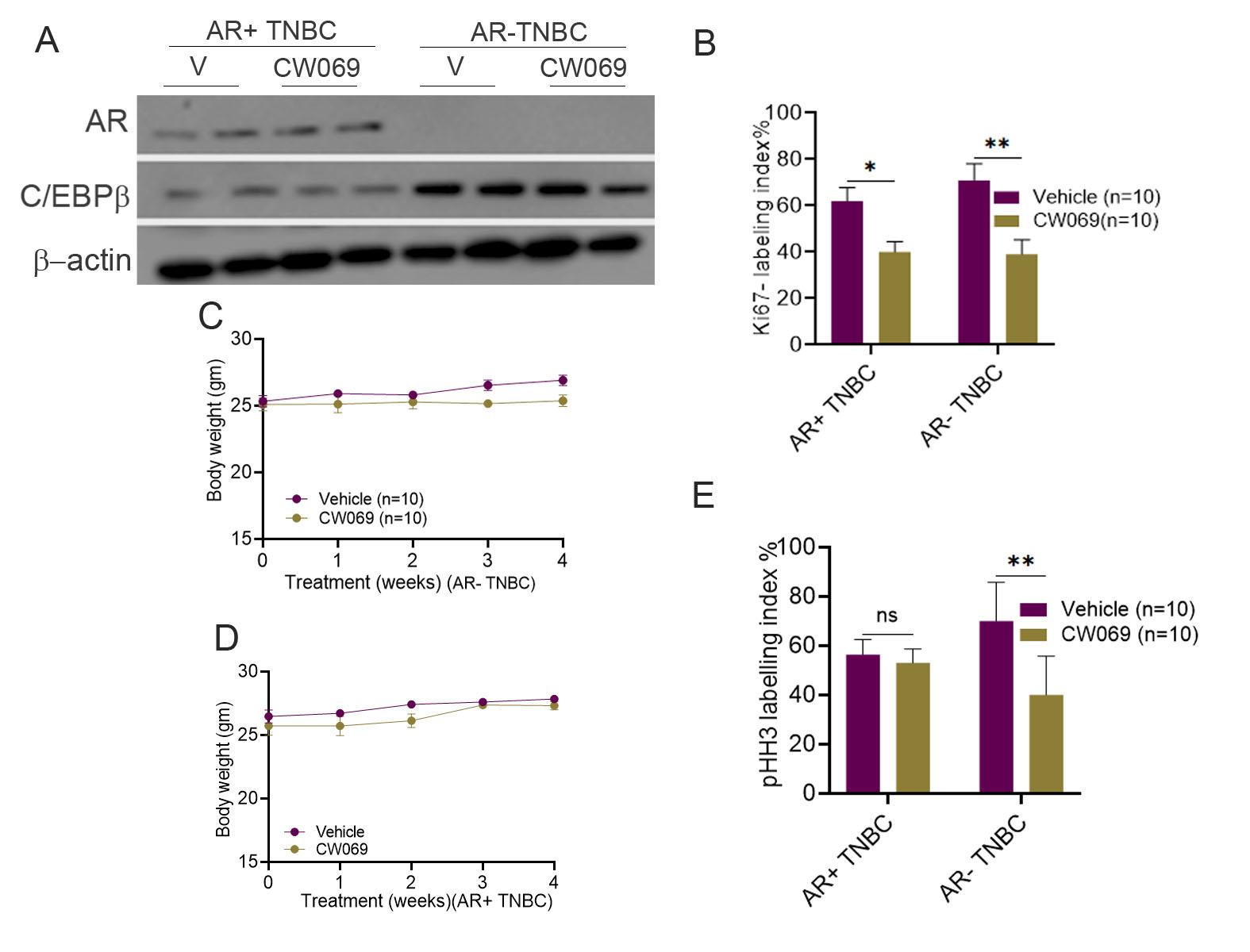


**Suppl. Fig. 5: CW069 treatment reduces tumor growth in mice bearing AR-TNBC tumors.** **(A)** Immunoblot showing the protein levels of AR and C/EBPβ in vehicle- and CW069-treated mice with AR+TNBC and AR-TNBC tumors. **(B, C)** Bar graphs showing Ki-67 **(B)** and pHH3 **(C)** expression in vehicle- and CW069-treated mice with AR+TNBC and AR-TNBC tumors. (**D, E**) Graphs showing the body weights of untreated and CW069-treated mice bearing AR+TNBC (**D**) and AR-TNBC (**E**) tumors. Bars indicate mean ± SEM. Unpaired two-tailed Student’s *t*-test with Welch’s correction was used to determine statistical significance. ***P*< 0.005, ns = non-significant.

**Tables**

**Table 1. Patient characteristics**

|  | **AR+TNBC  (n=24)** | **AR-TNBC  (n=58)** |
| --- | --- | --- |
| **AR status** |  |  |
| Positive | 21 | 0 |
| Negative | 0 | 58 |
| Unknown | 2 | 0 |
| **Age** |  |  |
| ≤60 | 7 | 26 |
| >60 | 16 | 32 |
| **Race** |  |  |
| White American | 15 | 9 |
| Black American | 4 | 38 |
| Unknown | 5 | 11 |
| **Chemo type** |  |  |
| Adjuvant | 17 | 40 |
| No treatment | 6 | 18 |
| **Tubule formation** |  |  |
| ≤2 | 2 | 9 |
| >2 | 21 | 49 |
| **Nuclear pleomorphism** |  |  |
| ≤2 | 5 | 7 |
| >2 | 18 | 51 |
| **Mitosis** |  |  |
| ≤5 | 19 | 49 |
| >5 | 4 | 9 |
| **Nottingham score** |  |  |
| ≤5 | 4 | 11 |
| >5 | 19 | 47 |
| **Nottingham grade** |  |  |
| 1 | 4 | 1 |
| 2 | 3 | 5 |
| ≥3 | 16 | 52 |
| **Tumor size (cm)** |  |  |
| ≤3 | 13 | 43 |
| >3 | 10 | 15 |
| **Vital status** |  |  |
| Alive | 19 | 21 |
| Dead | 4 | 37 |
| **Recurrence** |  |  |
| Yes | 0 | 5 |
| No | 21 | 45 |
| Unknown | 2 | 8 |
| **Invasion** |  |  |
| Yes | 0 | 10 |
| No | 21 | 38 |
| Unknown | 2 | 10 |

**Table 2.** List of reagents and antibodies

| **Name of reagent/antibody** | **Catalog no.** | **Company** |
| --- | --- | --- |
| Lipofectamine RNAiMAX | 13778075 | ThermoFisher Scientific |
| KIFC1 siRNA | L-004958-00-0005 | Horizon Discovery |
| KIFC1 antibody | ab172620 | Abcam |
| RNAeasy mini kit | 74104 | Qiagen |
| iScript cDNA synthesis kit | 1708891 | Bio-Rad |
| SSO advanced universal SYBR green supermix | 1725271 | Bio-Rad |
| Protease inhibitor cocktail | P8340 | Sigma Aldrich |
| AR antibody | M356201-2 | Dako |
| C/EBPβ antibody | NBP2-37567 | Novus biologicals |
| Androgen Receptor (AR) (NM_001011645) Human Tagged ORF Clone | RC215709 | OriGene |
| β-actin antibody | SC-47778 | Santa Cruz Biotechnology |
| Goat anti-mouse HRP antibody | SC-2005 | Santa Cruz Biotechnology |
| Goat anti-rabbit HRP antibody | 4050-05 | SouthernBiotech |
| ECL kit | 32106 | Thermo Scientific |
| BrdU cell proliferation kit | 2750 | EMD Millipore |
| BrdU antibody | ab152095 | Abcam |
| α tubulin antibody | T9026 | Sigma Aldrich |
| CW069 | HY-15857 | MedChem Express |
| ChIP enzymatic kit | 9003 | Cell signaling technology |
| C/EBPB siRNA | L-006423-00-0005 | Horizon Discovery |
| 1X phosphate buffer saline | MT21040CV | Corning |
| AR siRNA | L-003400-00-0005 | Horizon Discovery |
| AR ChIP grade antibody | 5153 | CST |
| C/EBPB ChIP grade antibody | PA5-27244 | Invitrogen |
| 37% paraformaldehyde | 252549-500ML | Millipore Sigma |
| Crystal violet | C0775-25G | Millipore Sigma |
| Diva 10X antigen retrieval buffer | DV2004LX | Biocare Medical |
| Ki-67 antibody | CRM325C | Biocare Medical |
| pHH3 antibody | 3130 | Biocare Medical |
| Rabbit HRP antibody | RHRP520L | Biocare Medical |
| Mouse HRP antibody | MHRP520L | Biocare Medical |
| Nude (nu/nu) mice | 002019 | Jackson laboratories |
| Maxi prep kit | 12165 | Qiagen |
| Lipofectamine LTX plus | 15338030 | Thermo Fisher Scientific |
| CellTiter-Glo® 2.0 Cell Viability kit | G9242 | Promega |
| Epithelial-Mesenchymal Transition (EMT) Antibody Sampler Kit | 9782T | Cell Signaling Technology |
| Cell Cycle Regulation Antibody Sampler Kit | #9932 | Cell Signaling Technology |
| MUC1 antibody | ab15481 | Abcam |
| MMP-9 antibody | #3852 | Cell Signaling Technology |
| TWIST1 (E7E2G) antibody | #69366 | Cell Signaling Technology |
| Cyclin B1 antibody | #4138 | Cell Signaling Technology |
| HMGA1 (D6A4) antibody | #7777 | Cell Signaling Technology |
| Cyclin A2 (BF683) | #4656 | Cell Signaling Technology |
| Cyclin E antibody | #4129 | Cell Signaling Technology |

**Table 3.** List of qRT-PCR primers

| Target gene | Forward primer sequence | Reverse primer sequence |
| --- | --- | --- |
| *AR* | GTGCTGGACACGACAACAAC | GATCAGGGGCGAAGTAGAGC |
| *KIFC1* | CGTGCGAGTTCTCTACCCTG | CAGCCTCCTCTCCTTCTCCT |
| *CEBPβ* | AAGCACAGCGACGAGTACAA | ACAGCTGCTCCACCTTCTTC |
| *CCND1* | TACCGTTGACTTCCAGGCAC | GACAGACAAAGCGTCCCTCA |
| *CCNA2* | ACCCAGGGTTCTCAGAATGG | CTTGGATGCCAGTCTTACTCA |
| *CDK6* | CCGACTGACACTCGCAGC | GACTTCGGGTGCTCTGTACC |
| *CDK4* | TGAAATTGGTGTCGGTGCCT | TGGTCGGCTTCAGAGTTTCC |
| *CDH1* | TGGTACCTGGCAAGATGCAG | GGGGGCTTCATTCACATCCA |
| *Vim* | GGACCAGCTAACCAACGACA | AAGGTCAAGACGTGCCAGAG |
| *Snai1* | CGAGTGGTTCTTCTGCGCTA | GGGCTGCTGGAAGGTAAACT |
| *Slug* | CTCCTCATCTTTGGGGCGAG | TCCTTGAAGCAACCAGGGTC |
| *Twist* | CGGCCAGGTACATCGACTTC | CAGAGGTGTGAGGATGGTGC |
| *Zeb1* | GCTGTTTCAAGATGTTTCCTTCCA | TTACACCCAGACTGCGTCAC |
| *MMP9* | TCTATGGTCCTCGCCCTGAA | TTGTATCCGGCAAACTGGCT |
| *MUC1* | CCTCACAGTGCTTACAGTTGTT | AGTAGTCGGTGCTGGGATCT |
| ARE-*C/EBPβ* proximal (for ChIP) | GGCCGCCCTTATAAATAACC | TATTAGTGAGGGGGCTGGTG |
| ARE*-MYC*-ChIP | GCTCTGGGCACACACATTGG | GGCTCACCCTTGCTGATGCT |
| CEBPB-RE *KIFC1*  (-598) ChIP | CCAGCAGTGTGACCTTATTGTG | CGAGCTCGGGGAAGATTTACT |

**Table 4.** Genes negatively correlated with *AR.* Source: UALCAN

| Gene | Pearson correlation |
| --- | --- |
| *SNRPA* | -0.39 |
| *RPP21* | -0.38 |
| *MIIP* | -0.38 |
| *CEBPB* | -0.37 |
| *POLR2F* | -0.37 |
| *COX4I1* | -0.37 |
| *RPS19* | -0.36 |
| *PSMG3* | -0.36 |
| *LSM7* | -0.35 |
| *SHFM1* | -0.35 |
| *C19orf43* | -0.35 |
| *RPS19BP1* | -0.34 |
| *FAM96B* | -0.34 |
| *FAM100B* | -0.34 |
| *NDUFS5* | -0.34 |
| *DGUOK* | -0.34 |
| *UQCRH* | -0.33 |
| *FAM58A* | -0.33 |
| *PFN1* | -0.33 |
| *MRPS24* | -0.33 |
| *MFSD2B* | -0.33 |
| *SH3BGRL3* | -0.33 |
| *CYBA* | -0.33 |
| *TSPO* | -0.33 |
| *PSMG4* | -0.33 |
| *EIF5A* | -0.33 |
| *RPL13* | -0.33 |
| *PFDN2* | -0.33 |
| *ZNHIT1* | -0.33 |
| *RPLP2* | -0.33 |
| *CCDC107* | -0.33 |
| *NUDT1* | -0.32 |
| *C9orf16* | -0.32 |
| *TUBB6* | -0.32 |
| *SURF2* | -0.32 |
| *C6orf108* | -0.32 |
| *BAX* | -0.32 |
| *TMUB1* | -0.32 |
| *BRI3* | -0.32 |
| *GADD45GIP1* | -0.32 |
| *RPL35* | -0.32 |
| *NDUFA11* | -0.32 |
| *C16orf61* | -0.32 |
| *SRM* | -0.31 |
| *CCDC124* | -0.31 |
| *GUK1* | -0.31 |
| *ATP5J2* | -0.31 |
| *ATAD3B* | -0.31 |
| *ZNF593* | -0.31 |
| *C21orf70* | -0.31 |
| *MUTYH* | -0.31 |
| *PUS1* | -0.31 |
| *RRP1* | -0.31 |
| *UBE2S* | -0.3 |
| *TIMM16* | -0.3 |
| *CTU2* | -0.3 |
| *MPST* | -0.3 |
| *ST20* | -0.3 |
| *C7orf55* | -0.3 |
| *RPS7* | -0.3 |
| *CAMTA1* | -0.3 |
| *UQCRHL* | -0.3 |
| *NCRNA00116* | -0.3 |
| *AURKB* | -0.3 |
| *AURKAIP1* | -0.3 |
| *C9orf142* | -0.3 |
| *ATP6V1F* | -0.3 |
| *CCDC102A* | -0.3 |
| *YDJC* | -0.3 |
| *ATAD3A* | -0.3 |
| *BCL7C* | -0.3 |
| *LIN37* | -0.3 |
| *PLEKHO1* | -0.3 |
| *EXOSC4* | -0.3 |
